# Biomarkers for early detection and monitoring of abnormal brain development in mild fetal growth restriction

**DOI:** 10.1016/j.isci.2025.113237

**Published:** 2025-07-30

**Authors:** Atsuto Onoda, Yuma Kitase, Jacques-Olivier Coq, Kazuto Ueda, Shinobu Shimizu, Masahiro Tsuji, Masahiro Hayakawa, Yoshiaki Sato

**Affiliations:** 1 Division of Neonatology, Center for Maternal-Neonatal Care, Nagoya University Hospital, Nagoya 466-8560, Japan; 2 Faculty of Pharmaceutical Sciences, Sanyo-Onoda City University, Sanyo-Onoda City 756-0884, Japan; 3 Centre National de la Recherche Scientifique (CNRS), Institut des Sciences du Mouvement (ISM) UMR7287, Aix Marseille Université, Marseille Cedex 09, France; 4 Department of Advanced Medicine, Nagoya University Hospital, Nagoya 466-8560, Japan; 5 Department of Food and Nutrition, Kyoto Women’s University, Higashiyama-ku, Kyoto 605-8501, Japan

**Keywords:** Neuroscience, Developmental biology, Omics

## Abstract

Fetal growth restriction (FGR), driven by intrauterine hypoperfusion, delays brain development and heightens the risk of neurodevelopmental disorders. Nonetheless, current diagnostic strategies rarely capture the subtle neuropathology that emerges in mild FGR. To overcome this limitation, we employed an innovative rodent model that replicates mild FGR through gradual and chronic intrauterine hypoperfusion, mirroring clinical conditions overlooked by conventional severe or acute FGR models. Global proteomics of cerebrospinal fluid identified Alpha-2-Macroglobulin, Neuroserpin, CD200, and Polyubiquitin-B as biomarkers correlated with birth weight and persisting postnatally. Their expression reflected changes in brain tissue and serum, was associated with behavioral deficits, and partially recovered under mesenchymal stem/stromal cell treatment—indicating potential for therapeutic monitoring. Notably, brain-specific Neuroserpin, emerged as a robust indicator of FGR-related neurodevelopmental impairment. This study is the first to propose low-invasive serum biomarkers for the early postnatal detection of mild FGR-induced brain abnormalities, enabling neonatal screening, targeted interventions, and improved long-term outcomes.

## Highlights

• A rat model of gradual chronic hypoperfusion reliably mimics mild clinical FGR
• CSF proteomics identifies four birthweight biomarkers: Neuroserpin, A2M, CD200, and Ubb
• Biomarker changes post-MSC therapy serve as indicators of treatment efficacy
• Serum Neuroserpin enables minimally invasive screening of neuro-risk in mild FGR

## Graphical abstract

**Figure:**
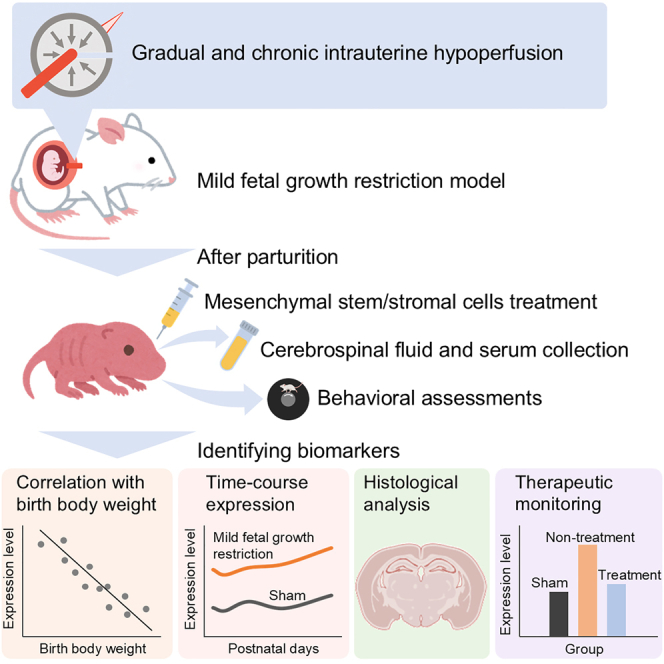

## Introduction

In medical science, developing preventative therapeutic strategies for central nervous system disorders is widely recognized as one of the predominant challenges. Globally, growing concerns have emerged over the rise in children with neurodevelopmental disorders (NDDs), such as autism spectrum disorder (ASD), attention-deficit/hyperactivity disorder (ADHD), and learning disabilities. For instance, the prevalence of ASD or ADHD among children in the United States increased from 6.3% to 10.8% between 1997 and 2021.^1^ Considering the presence of undiagnosed and potential NDDs, these figures are likely underestimated, suggestive of a higher prevalence than reported.^2^ Consequently, there is an urgent need to improve outcomes and implement early preventive measures for NDDs to safeguard the health of future generations.

Fetal growth restriction (FGR), also known as intrauterine growth restriction (IUGR), has been identified as a significant risk factor for NDDs.^3^^,^^4^ FGR, which refers to the cessation or deceleration of fetal growth *in utero*, is a primary cause of low birth weight.^5^ FGR accounts for 5–10% of prenatal occurrences, affecting an estimated 30 million fetuses globally yearly.^6^^,^^7^^,^^8^ Although potential risk factors such as environmental pollutants and advanced maternal age have been suggested, the direct causes of FGR remain unclear.^9^ Chronic insufficiency of uterine blood flow, which leads to hypoxemia and nutritional deficiency, commonly underlies FGR.^4^^,^^10^ Chronic intrauterine hypoperfusion adversely impacts brain development, thereby elevating the likelihood of subsequent NDDs.^5^^,^^11^^,^^12^

Critically, even mild cases of FGR, with no overt postnatal imaging abnormalities, can later manifest as NDDs in infancy or childhood.^13^^,^^14^^,^^15^ Although severe FGR is typically recognized clinically, mild cases may be overlooked, potentially leading to unrecognized risks of lifelong neurological deficits. Despite the urgent need for preventive and therapeutic strategies for brain developmental abnormalities caused by FGR, current diagnostic technologies often fail to detect subtle, early-stage brain abnormalities in each FGR infant. Early and accurate diagnosis could facilitate therapeutic interventions during the neonatal period, when neuroplasticity is highest and treatment responsiveness is the most favorable. It also assists families in creating optimal caregiving environments as soon as possible. Accordingly, foundational and translational research in neonatal medicine has increasingly focused on identifying early diagnostic markers for NDDs associated with FGR. Although the direct evaluation of mild FGR in human neonates is ideal, it poses ethical and logistical challenges—particularly for cerebrospinal fluid (CSF) sampling. Therefore, we employed a recently developed animal model that replicates mild chronic intrauterine hypoperfusion, which more closely reflects the gradual blood flow insufficiency observed clinically.^16^^,^^17^ This approach contributes to generating insights that can ultimately inform human diagnostics and interventions.

In this study, we conducted comprehensive proteomic analyses of postnatal rat CSF to discover potential biomarkers. We then examined their expression in postnatal brain tissue and serum to determine whether these molecules could serve as minimally invasive blood-based indicators of brain abnormalities. Furthermore, we investigated whether these putative biomarkers could monitor therapeutic efficacy—specifically under mesenchymal stem/stromal cell (MSC) treatment—thereby underscoring their broader clinical applicability. By incorporating behavioral assessments, we aimed to establish a multi-layered validation approach that could, in the future, guide the development of low-invasive, serum-based diagnostic tools for mild FGR in humans.

## Results

### Mild fetal growth restriction is replicated by gradual and chronic intrauterine hypoperfusion

**Figure 1:**
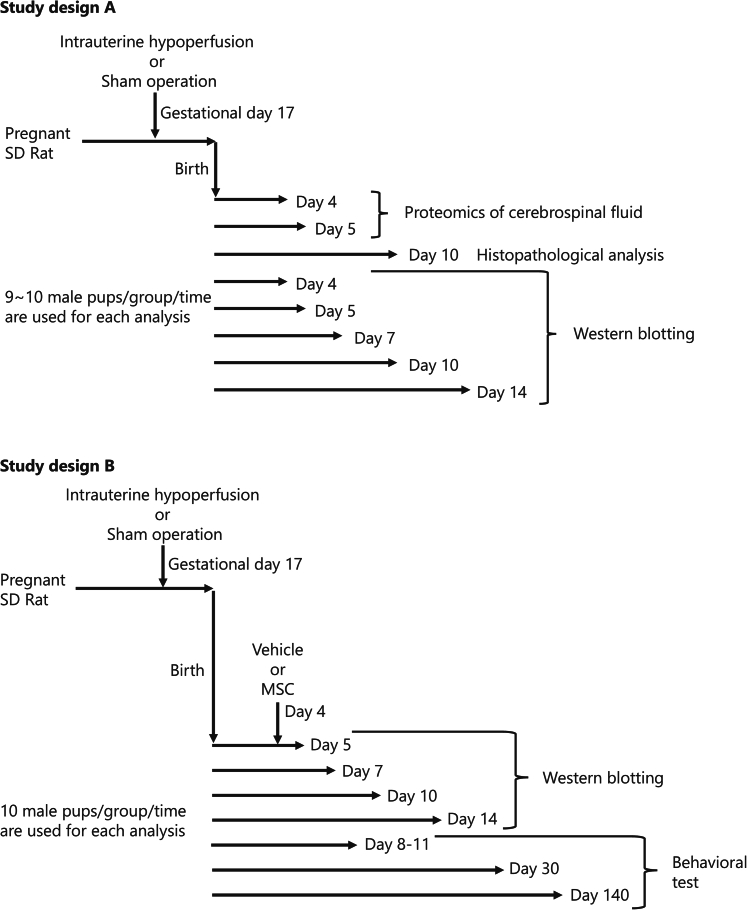
Experimental design (A) Study Design A: Intrauterine hypoperfusion surgery to induce fetal growth restriction was performed on gestational day 17. After birth, cerebrospinal fluid was collected from the offspring for comprehensive protein analysis. The identified biomarker candidates were further analyzed for their time-course expression changes up to postnatal day 14, as well as histological changes in brain tissue at postnatal day 10. (B) Study Design B: Model animals were created in the same way as those in study design A. On postnatal day 4, mesenchymal stem/stromal cells (MSCs) or vehicle were injected into the offspring. Subsequently, cerebrospinal fluid and serum were collected to evaluate the expression changes of biomarker candidates. Additionally, brain functions were assessed through behavioral tests.

**Figure 2:**
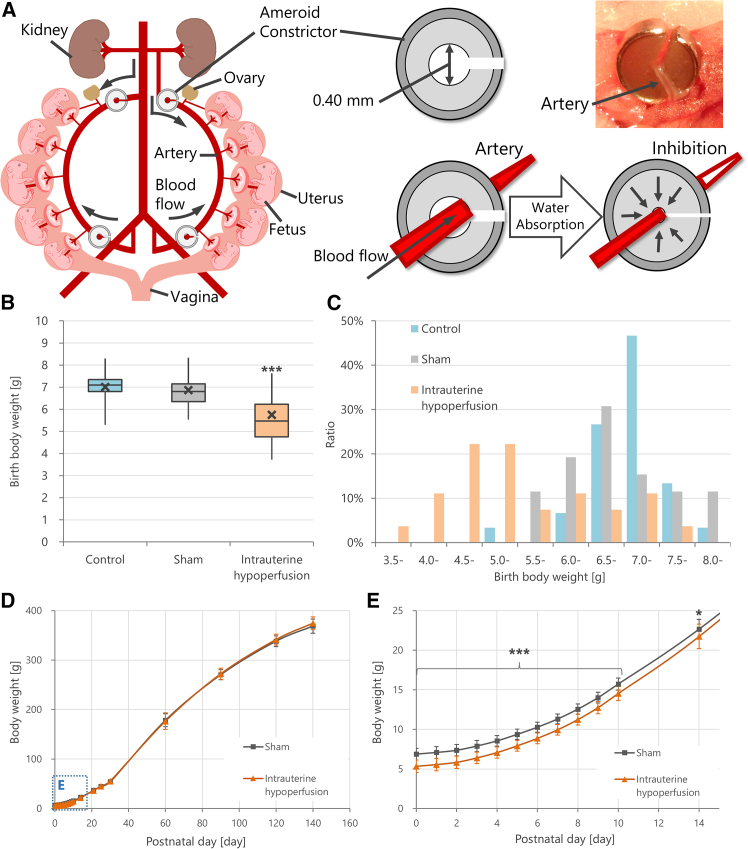
Creation of fetal growth restriction model and body weight phenotype (A) Schematic of the intrauterine hypoperfusion model using an ameroid constrictor, which gradually reduces uterine arterial blood flow by absorbing water and causing mechanical compression, resulting in mild chronic fetal hypoperfusion. The image on the right shows the actual placement of the constrictor around the uterine artery. (B) Birth weights of the Control (non-treated), Sham (surgery without hypoperfusion), and Intrauterine hypoperfusion (ameroid constrictor-attached) groups. Data are presented using box plots, showing the interquartile range with means indicated by cross marks (n = 26–30/group, biological replicates, ∗∗∗p < 0.001, Steel-Dwass test). The hypoperfusion group showed significantly lower birth weights. (C) Histograms of birth weights in each group. The threshold for defining fetal growth restriction (FGR), based on the mean ± standard deviation (SD) of the Control group, was 6.18 g. In the hypoperfusion group, 73% of offspring fell below this threshold and were classified as FGR models. (D and E) Body weight trajectories from postnatal day 0–140. Data are presented as mean ± SD (n = 26–30/group, biological replicates, ∗∗∗p < 0.001, ∗p < 0.05 by Wilcoxon signed-rank test). (E) Enlarged view from postnatal day 0–25. The hypoperfusion group showed significant weight reduction until postnatal day 14, followed by catch-up growth.

In the clinical presentation, FGR varies in phenotype depending on its severity. When conducting studies using FGR model animals, the methodology of model creation is critical, and the degree of severity of FGR being reproduced must be considered. In the present study, we used an animal model replicating mild cases of FGR, achieved by gradually and chronically restricting intrauterine blood flow using a hydrophilic vessel constrictor known as an ameroid constrictor (Figure 1, Figure 2A).^16^^,^^17^ This animal model showed phenotypes similar to clinical observations in mild cases of FGR children,^18^^,^^19^ characterized by minimal structural damage and only mild reductions in the number of cerebral cortical neurons. Importantly, behavioral phenotypes such as impaired motor coordination and diminished primitive reflexes were pronounced, reflecting key clinical features observed in mild cases of FGR.

Not all offspring born to dams subjected to mild intrauterine hypoperfusion exhibited FGR. Therefore, in this study, FGR offspring were identified based on their birth weight. International clinical criteria for FGR typically define it as fetal weight below the 3rd to 10th percentiles, which corresponds to −1.28 to −1.88 standard deviations (SD) in a normally distributed population.^9^^,^^20^ Notably, fetuses with weights below the 5th percentile, equivalent to −1.64 SD, show a higher incidence of perinatal complications. Based on these findings, it is recommended that a value equivalent to −1.5 SD from the fetal weight standards be used as a criterion for FGR diagnosis in Japan.^21^ Although fetal weight and birth weight are not strictly identical, they are considered clinically comparable. In this study, fetal weight measurement in experimental animals was difficult; therefore, we adopted −1.5 SD of the untreated group’s mean birth weight as the threshold for the FGR model.

The birth weight of the untreated group (Control group) in the present study was 7.01 ± 0.55 g (Figure 2B), and the FGR model threshold was calculated to be 6.18 g. Individuals with birth weights below this threshold were designated as FGR models. Post-surgical uterine blood flow insufficiency (intrauterine hypoperfusion group) resulted in 73% of the offspring falling below this FGR model threshold (Figure 2C). In contrast, the sham-operated group (Sham group), in which only laparotomy and uterine exposure were performed without the installation of the ameroid constrictors, had a birth weight of 6.87 ± 0.74 g, with 15% falling below the FGR model threshold (Figures 2B and 1C). When comparing the weight trajectories between the Sham and intrauterine hypoperfusion group, a significant difference was observed from postnatal day (PND) 0–14. After day 14, which they caught up, and no significant difference was noted (Figures 2D and 1E), suggesting that the low birth weight associated with mild intrauterine hypoperfusion procedure was maintained until approximately two weeks after birth.

### Cerebrospinal fluid profiling identifies six proteins as promising fetal growth restriction biomarker candidates

To identify biomarkers reflecting brain developmental abnormalities associated with FGR, the present study conducted a comprehensive analysis of proteins in CSF. CSF, a tissue fluid circulating within the brain, contained various biomolecules secreted by brain cells.^22^ The types and quantities of these biomolecules depended on the condition of the brain, making the CSF a potential source of biomarker candidates that sensitively reflected the brain’s status.^23^ Indeed, previous studies have demonstrated the utility of analyzing CSF molecules in the search for diagnostic biomarkers in central nervous system diseases such as Alzheimer’s disease.^24^

**Figure 3:**
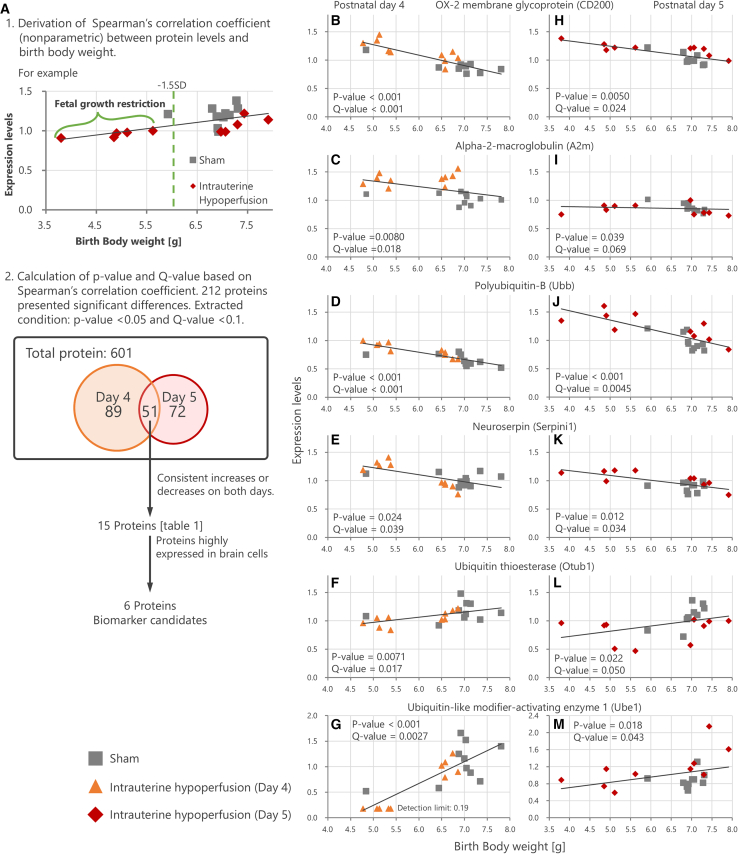
Comprehensive analysis of proteins in cerebrospinal fluid (A) Analysis process of the cerebrospinal fluid protein profile and extraction conditions for biomarker candidates. A total of 212 proteins (51 duplicates) showed significant changes proportional to the decrease in birth weight: 140 proteins on postnatal day 4 and 123 proteins on the postnatal day 5 (*p*-value <0.05 and Q-value <0.1 by Spearman’s rank correlation coefficient and Storey’s method). (B–M) Correlation plots showing the relationship between the expression levels of identified biomarker candidates and birth body weight on postnatal day 4 (left) and day 5 (right) in the intrauterine hypoperfusion and sham groups. *p*-values, and Q-values are presented in each panel.

In our study, CSF was collected from offspring on days 4 and 5 after birth (Figure 1). Liquid chromatography-tandem mass spectrometry (LC-MS/MS) was used to quantify all proteins, thereby obtaining a protein profile. For all 601 proteins detected and quantified from the CSF, we calculated the Spearman’s rank correlation coefficient with the body weight at birth of the rat pups, as well as the *p*-value and False Discovery Rate (Storey’s method Q-value) based on this correlation coefficient. Proteins showing significant expression changes associated with low birth weight were selected as biomarker candidates under the criteria of *p*-value <0.05 and Q-value <0.1 (Figure 3A). A total of 212 proteins were identified as changing in association with low birth weight, with 140 detected on PND 4 and 123 on PND 5, including 51 overlapping between the two days. We selected only those among the 51 proteins that showed consistent significant changes on both PNDs 4 and 5 (either increase or decrease on both days), resulting in 15 molecules being identified (Table 1).

**Table 1:** List of biomarker candidate molecules for abnormal brain development induced by fetal growth restriction

Uniprot Accession		P04218	P06238	P0CG51	Q9JLD2	B2RYG6	Q5U300
Symbol		Cd200	A2m	Ubb	Serpini1	Otub1	Ube1
Description		OX-2 membrane glycoprotein	Alpha-2-macroglobulin	Polyubiquitin-B	Neuroserpin	Ubiquitin thioesterase OTUB1	Ubiquitin-like modifier-activating enzyme 1
MW [kDa]		31.068	163.682	34.347	46.248	31.250	117.713
Day 4	coefficient	−0.81	−0.58	−0.87	−0.50	0.58	0.71
	*p*-value	0.000017	0.0080	0.0000006	0.0240	0.0072	0.0004
	Q-value	0.00029	0.0182	0.000039	0.039	0.017	0.0027
Day 5	coefficient	−0.60	−0.46	−0.74	−0.55	0.51	0.52
	*p*-value	0.0050	0.039	0.0002	0.012	0.022	0.018
	Q-value	0.024	0.069	0.0045	0.035	0.050	0.043
Brain location	Cerebral cortex	Neuronal cell (High)	Glial cell (High)	Neuronal cell (High)	Neuronal cell (High)	Endothelial cell (High)	Neuronal cell (Medium)
		Glial cell (Medium)	Neuronal cell (Medium)	Neuropil (Low)	Neuropil (Medium)	Neuronal cell (Low)	Glial cell (Medium)
		Neuropil (Low)	Endothelial cell (Medium)		Endothelial cell (Low)		Endothelial cell (Low)
			Neuropil (Low)				Neuropil (Low)
	Hippocampus	Neuronal cell (Medium)	Neuronal cell (Medium)	Neuronal cell (Medium)	Neuronal cell (Medium)	Neuronal cell (Low)	Glial cell (Low)
			Glial cell (Low)				Neuronal cell (Low)
	Caudate	Neuronal cell (Low)	Neuronal cell (Medium)	Neuronal cell (Low)	Neuronal cell (Medium)	Not detected	Glial cell (Low)
			Glial cell (Low)				Neuronal cell (Low)
	Cerebellum	Cell in molecular layer (Low)	Granular layer (High)	Cell in molecular layer (Low)	Purkinje cell (Medium)	Purkinje cell (Low)	Purkinje cell (High)
		Cell in granular layer (Low)	Molecular layer (High)	Cell in granular layer (Low)	Granular layer (Medium)		Granular layer (Medium)
		Molecular layer (Low)	Purkinje cell (Medium)	Purkinje cell (Low)	Molecular layer (Medium)		Molecular layer (Medium)

Uniprot Accession		Q5ZQU0	Q9ET61	P07483	P09006	P15429	Q9QX79	Q99PS8	P08649	P10959
Symbol		Sned1	Cd93	Fabp3	Serpina3	Eno3	Fetub	Hrg	C4	Ces1
Description		Sushi, nidogen	Complement component C1q receptor	Fatty acid-binding protein, heart	Serine protease inhibitor A3N	Beta-enolase	Fetuin-B	Histidine-rich glycoprotein	Complement C4	Carboxyl-esterase 1C
MW [kDa]		151.301	68.737	14.766	46.622	46.984	41.536	59.012	192.042	60.136
Day 4	coefficient	0.75	0.48	0.53	−0.53	0.48	−0.54	0.46	−0.54	0.57
	*p*-value	0.00016	0.0317	0.015	0.016	0.034	0.013	0.040	0.0132	0.0093
	Q-value	0.0014	0.047	0.028	0.029	0.050	0.025	0.055	0.025	0.019
Day 5	coefficient	0.50	0.51	0.57	−0.70	0.54	−0.57	0.57	−0.86	0.71
	*p*-value	0.025	0.023	0.0093	0.00066	0.013	0.0091	0.0092	0.0000015	0.00050
	Q-value	0.053	0.049	0.032	0.0080	0.037	0.032	0.032	0.00014	0.0074
Brain location	Cerebral cortex	Endothelial cell (Low)	Endothelial cell (Low)	Not detected	Not detected	Not detected	Not detected	Not detected	Not detected	Not detected
		Neuronal cell (Low)								
	Hippocampus	Glial cell (Low)	Not detected	Neuronal cell (Low)	Not detected	Not detected	Not detected	Not detected	Not detected	Not detected
		Neuronal cell (Low)								
	Caudate	Not detected	Not detected	Not detected	Not detected	Not detected	Not detected	Not detected	Not detected	Not detected
	Cerebellum	Purkinje cell (Low)	Not detected	Not detected	Molecular layer (Low)	Not detected	Not detected	Not detected	Not detected	Not detected
		Granular layer (Low)								

In the cerebrospinal fluid protein profiles, the molecules that were consistently increased or decreased on both days 4 and 5 of birth were tabulated. The table includes the correlation coefficients, *p*-values, and Q-values obtained from the protein profile analysis, as well as the Uniprot Accession, molecular weight (MW), and brain localization of each molecule. Brain localization was referenced from the Human Protein Atlas database (https://www.proteinatlas.org/). Among the 15 molecules, six molecules, namely OX-2 membrane glycoprotein (CD200), α-2-macroglobulin (A2m), Polyubiquitin-B (Ubb), Neuroserpin (Serpini1), Ubiquitin thioesterase OTUB1 (Otub1), and Ubiquitin-like modifier-activating enzyme 1 (Ube1), were confirmed to be highly expressed in brain cells.

Among these 15 molecules, OX-2 membrane glycoprotein (CD200), α-2-macroglobulin (A2m), Polyubiquitin-B (Ubb), Neuroserpin (Serpini1), Ubiquitin thioesterase OTUB1 (Otub1), and Ubiquitin-like modifier-activating enzyme 1 (Ube1) were confirmed to exhibit high expression in the brain cells according to the Human Protein Atlas database (https://www.proteinatlas.org), and were selected as biomarker candidates (Table 1; Figures 3B–3M). Although other proteins (e.g., Sned1 and Cd93) were also detected as differentially expressed in the CSF proteomics, we deprioritized them for validation because the Human Protein Atlas database and existing literature indicated low or negligible brain expression. Our primary focus was on proteins with established or predicted CNS involvement, as our overarching goal is to identify serum biomarkers that leak from the CSF and reflect brain-specific or brain-predominant processes relevant to FGR-related neurodevelopmental deficits.

### Neuroserpin, OX-2 membrane glycoprotein, polyubiquitin-B, and α-2-macroglobulin show sustained two-week expression

**Figure 4:**
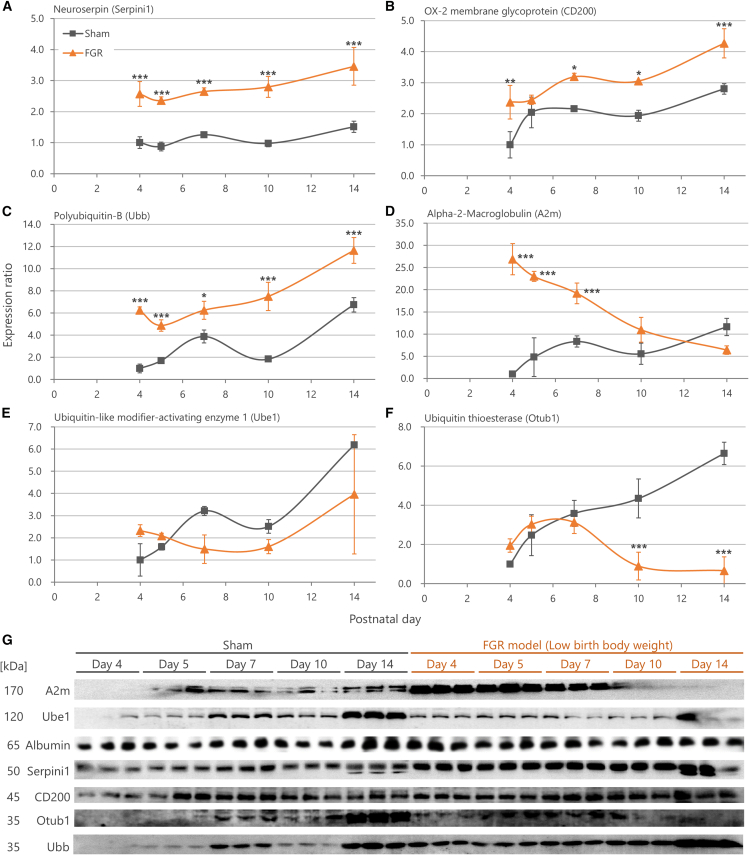
Time-course expression changes of biomarker candidates in cerebrospinal fluid (A–F) Graphs showing expression changes of each molecule from postnatal day 4 to day 14. Relative expression levels were shown with the Sham group on day 4 set as 1. The relative expression levels of each data point were normalized by albumin, an endogenous control. Data are presented as mean ± SD (*n* = 15/group biological replicates, ∗∗∗*p* < 0.001, ∗∗*p* < 0.01, ∗*p* < 0.05 by Steel-Dwass test). (A) Neuroserpin (Serpini1), (B) OX-2 membrane glycoprotein (CD200), (C) Polyubiquitin-B (Ubb), (D) Alpha-2-Macroglobulin (A2m), (E) Ubiquitin-like modifier-activating enzyme 1 (Ube1), and (F) Ubiquitin thioesterase (Otub1). (G) Representative images of Western blotting bands.

To evaluate the potential diagnostic applications of the biomarker candidates, we conducted a time-course expression analysis in the CSF of rats from PNDs 4 to 14 using Western blotting (Figures 4A–4G). Assessing multiple time points allowed us to capture dynamic changes over time and identify biomarkers with stable and consistent expression profiles.

The results revealed that the expression changes in Neuroserpin were the most consistently maintained throughout the observation period (Figure 4A). Subsequently, CD200 and Ubb also showed sustained elevation in expression levels up to PND 14 (Figures 4B and 4C). Although the increased expression of A2m did not persist until day 14, it displayed significant expression changes from PNDs 4 to 7, suggesting its potential as a short-term biomarker (Figure 4D). Unlike these molecules, Ube1 exhibited no significant differences in expression between Sham and FGR groups at any of the tested time points, making it less likely to serve as a potential biomarker for FGR-related brain developmental abnormalities (Figure 4E). Meanwhile, Otub1 exhibited low expression levels with weak signals relative to background noise in the Western blot analysis (Figures 4F and 3G). This low signal-to-noise ratio made reliable detection more difficult, suggesting that Otub1 may not be a suitable biomarker despite the statistically significant expression changes observed at later time points (e.g., PNDs 10 and 14). Overall, these findings indicated that Neuroserpin, CD200, Ubb, and A2m have potential as promising biomarkers for abnormal brain development associated with FGR.

### Neuroserpin, α-2-macroglobulin, OX-2 membrane glycoprotein, and polyubiquitin-B are upregulated in distinct brain cell types in mild fetal growth restriction

**Figure 5:**
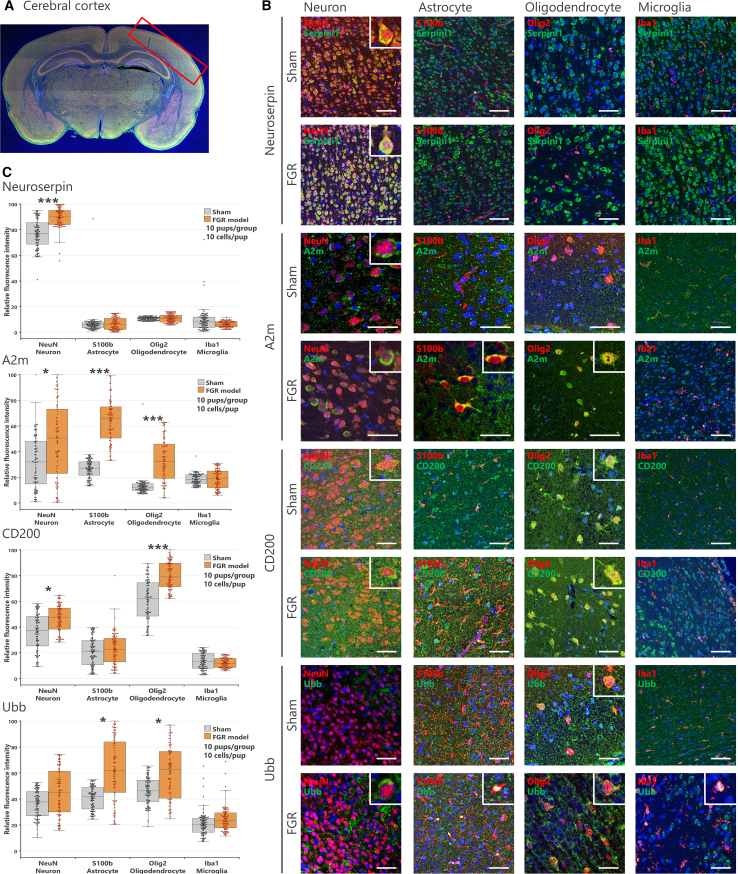
Localization and expression intensity of biomarker candidates in the cerebral cortex (A) Representative coronal section of the rat brain, indicating the analyzed region in the cerebral cortex (red box). (B) Immunofluorescence staining of biomarker candidate molecules in neurons (NeuN-positive cells), astrocytes (S100b-positive cells), oligodendrocytes (Olig2-positive cells), and microglia (Iba1-positive cells). Enlarged images of co-localized cells are shown in the upper right corner of each photo. Scale bar: 50 μm. (C) Graphs showing the relative fluorescence intensity of biomarker candidates in each cell. The highest intensity value was set at 100, and the background was set at 1. Data are shown as box-and-whisker plots depicting median (center line), first and third quartiles (boxes), and whiskers extending to the minimum and maximum values within 1.5× the interquartile range; dots represent individual data points (beeswarm), and outliers beyond the whiskers are shown separately. (*n* = 10/group, totaling 100 cells in each group, ∗∗∗*p* < 0.001, ∗∗*p* < 0.01, ∗*p* < 0.05 by Steel-Dwass test).

To confirm that the biomarker molecules detected in CSF were derived from brain cells, we performed histological analysis to evaluate the expression localization of each molecule in the brain at PND 10 (Figure 1). The expression localization and intensity of each biomarker candidate were evaluated in neurons (NeuN^+^), astrocytes (S100b^+^), oligodendrocytes (Olig2^+^), and microglia (Iba1^+^) in the cerebral cortex (Figure 5A), hippocampus (see Figure S1), and thalamus (see Figure S2).

Neuroserpin, which exhibits neuroprotective effects,^25^ was primarily expressed in neurons, with no expression observed in astrocytes, oligodendrocytes, or microglia (Figure 5B). Fluorescence intensity appeared higher in neurons in the FGR group compared to the Sham group, suggesting an increase in expression (Figure 5C). A2m, an acute phase response protein,^26^ was observed in some neurons in the Sham group, but not in other cell types. In contrast, in the FGR group, its expression was detected in neurons, astrocytes, and oligodendrocytes, with a notable increase in expression intensity in astrocytes and oligodendrocytes (Figure 5C). CD200, involved in microglial activation,^27^ was primarily observed in oligodendrocytes and neurons (Figure 5B). Its expression level in was significantly higher in the FGR group compared to the Sham group (Figure 5C). Ubb, which regulates in protein degradation,^28^ showed weak expression in the Sham group, but was expressed at high intensity in some astrocytes, and oligodendrocytes in the FGR group (Figure 5C).

The histological findings are consistent with the Western blotting results, suggesting that the observed increases in biomarker levels in the CSF may be attributed to specific cell types (Figure 4). The increase in Neuroserpin levels was likely due to elevated expression in neurons, while the rise in A2m levels corresponded to higher expression in astrocytes. Similarly, the increase in CD200 levels may be linked to oligodendrocyte expression, and the elevated Ubb levels reflected increased expression in neurons, astrocytes, and oligodendrocytes. The consistency between the Western blotting and immunohistochemistry results strengthens the reliability of these observed changes in biomarker expression across different cell types. Furthermore, results in the hippocampus (Figure S1) and thalamus (Figure S2) were similar to those observed in the cerebral cortex, suggesting that the expression of each molecule depends more on the type of cell rather than the brain region.

### Mesenchymal stem/stromal cell treatment ameliorates motor, reflex, and memory deficits associated with fetal growth restriction

For clinical applications as surrogate markers, it is essential to confirm consistent expression changes in response to pathological conditions. Accordingly, we specifically investigated whether the identified biomarkers would respond to the intravenous injection of MSCs in this FGR model, given their potential therapeutic implications.^16^^,^^29^

**Figure 6:**
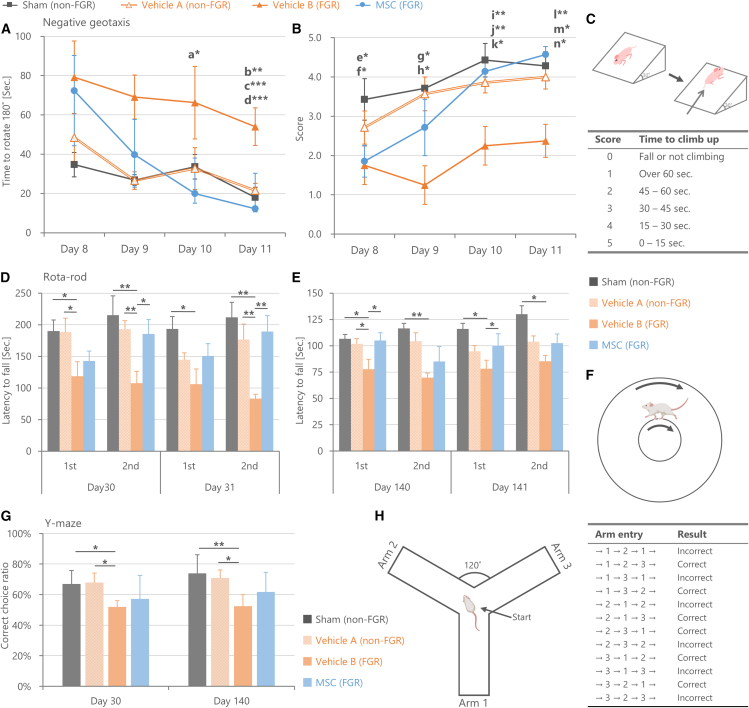
Behavioral assessments of brain function in the fetal growth restriction (FGR) model and effects of mesenchymal stem/stromal cells (MSCs) (A–C) Negative geotaxis test. (A) Time taken for pups to rotate 180° and face upward. (B) Climb-up score based on rotation time. (C) Schematic of the test and scoring criteria. The Vehicle B group (FGR: birth weights below the threshold) showed significant delays in turning over and reduced climb-up scores vs. Sham and Vehicle A (both non-FGR). These changes were improved by the MSC administration. Data are mean ± SD (n = 7–8/group/day; ∗∗∗*p* < 0.001, ∗∗*p* < 0.01, ∗*p* < 0.05 by Steel-Dwass test; a∗ Vehicle B vs. MSC, b∗∗ Vehicle B vs. Vehicle A, c∗∗∗ Vehicle B vs. Sham, d∗∗∗ Vehicle B vs. MSC, e∗ Sham vs. Vehicle B, f∗ Sham vs. MSC, g∗ Sham vs. Vehicle B, h∗ Sham vs. Vehicle A, i∗∗ Sham vs. Vehicle B, j∗∗ MSC vs. Vehicle B, k∗ Vehicle A vs. Vehicle B, l∗ MSC vs. Vehicle B, m∗∗ Sham vs. Vehicle B, n∗∗ Vehicle A vs. Vehicle B). (D–F) Rota-rod test: (D) Postnatal days 30–31, (E) postnatal days 140–141, and (F) Schematic. The Vehicle B group showed reduced walking times compared to Sham and Vehicle A, with no improvement on repeated trials; these deficits were mitigated by MSCs. Data are mean ± SD (n = 8–11/group/day, ∗∗*p* < 0.01, ∗*p* < 0.05). (G and H) Y-maze test. (H) Schematic and correct/incorrect arm choices. Inefficient exploration was seen in the Vehicle B group vs. Sham and Vehicle A, but improved with MSCs. Data are mean ± SD (n = 8–11/group/day, ∗∗*p* < 0.01, ∗*p* < 0.05).

Behavioral experiments were initially conducted to assess brain functions and the efficacy of MSCs for treating FGR-derived brain developmental abnormalities (Figure 6). Pups with surgically induced intrauterine hypoperfusion were divided into two: Vehicle A group with birth weights above the FGR model threshold (non-FGR) and Vehicle B group with birth weights below the threshold (FGR). On PND 4, MSCs (1×10^5^ cells/60 μL) were injected into the right jugular vein of FGR pups and vehicle solution were injected into the right jugular vein of FGR and non-FGR pups under 2% isoflurane anesthesia (Figure 1). Consequently, four experimental groups were compared: Sham–non-FGR–Vehicle, Intrauterine hypoperfusion–non-FGR–Vehicle (Vehicle A), Intrauterine hypoperfusion–FGR–Vehicle (Vehicle B), and Intrauterine hypoperfusion–FGR–MSCs.

The Negative geotaxis test, investigating primitive reflex response by assessing the time and posture of climbing a slope, was conducted on PNDs 8 to 11 (Figures 6A–6C). In the Vehicle A (non-FGR) group, no significant differences were observed, compared to the Sham group regarding latency and climb-up-score. In contrast, the Vehicle B (FGR) group exhibited a significant increase in time to turn over and a decrease in climb-up-score compared to both the Sham and Vehicle A (non-FGR) groups on all tested days, indicating impaired primitive reflex response. MSC administration on PND 4 to FGR pups significantly suppressed the decline in primitive reflex response associated with FGR. Although the MSC-treated group showed similar primitive reflex abilities to the Vehicle B group on PND 8, their abilities improved significantly on PNDs 10 and 11, reaching to the level comparable to that of the Sham group.

Furthermore, a Rota-rod test was conducted on PNDs 30–31 and 140–141 to evaluate sensorimotor coordination, mainly focusing on interlimb coordination (Figures 6D–6F). On PNDs 30 and 31, the results indicated that the Vehicle B (FGR) group showed a significantly shorter latency to fall down compared to the Sham and Vehicle A (non-FGR), suggesting a decline in motor coordination ability due to FGR (Figure 6D). A similar result was observed on PNDs 140 and 141 (Figure 6E). Typically, the latency to fall increases in the second trial due to motor learning. Whereas the Sham group showed a significant increase in walking duration between the first and second trials on both days 30–31 and days 140–141, such an increase was not observed in the Vehicle B (FGR) group on either day (Figures 6D and 6E). In contrast, there were no significant differences in walking duration between the Sham and Vehicle A (non-FGR) groups, indicating that Vehicle A did not show impairment in motor coordination ability. Importantly, MSC-treated group exhibited significantly longer walking durations compared to the Vehicle B (FGR) group on both PNDs 30 and 140 (*p* < 0.05), and their performance was comparable to that of the Sham group. These results suggest that MSCs ameliorated the decline in motor coordination ability associated with FGR.

Additionally, a Y-maze test indicated that the Vehicle B (FGR) group exhibited a significantly lower correct choice ratio compared to the Sham and Vehicle A (non-FGR) groups on both PNDs 30 and 140, indicating a decline in spatial memory (Figures 6G and 6H). MSC administration partially improved the correct choice ratio, bringing it to a level similar to that of the Sham group, suggesting that MSCs ameliorated short-term spatial memory caused by FGR (Figures 6G and 6H). Open field test and Novel object recognition test were also conducted to assess spontaneous movement and recognition memory, respectively; however, no significant differences were observed (see Figure S3).

### Mesenchymal stem/stromal cell administration restores fetal growth restriction-induced biomarker changes

**Figure 7:**
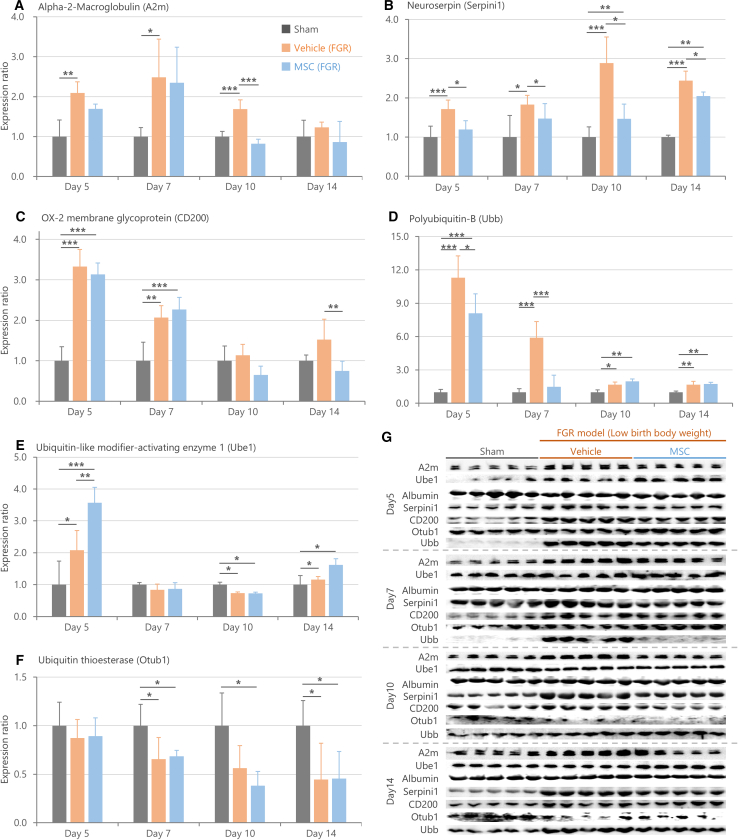
Treatment responsiveness of biomarker candidate molecules in cerebrospinal fluid (A–F) Relative expression levels of each molecule on postnatal days 5, 7, 10, and 14, with the Sham group set as 1 at each time point. The relative expression levels were standardized using albumin as an endogenous control. Data are presented as mean ± SD (*n* = 20/group biological replicates, ∗∗∗*p* < 0.001, ∗∗*p* < 0.01, ∗*p* < 0.05 by Steel-Dwass test). The levels of (A) Alpha-2-Macroglobulin (A2m), (B) Neuroserpin (Serpini1), (C) OX-2 membrane glycoprotein (CD200), and (D) Polyubiquitin-B (Ubb) were significantly changed in the Vehicle (FGR) group compared to the Sham group across multiple time points. Administration of mesenchymal stem/stromal cells (MSCs) on postnatal day 4 significantly suppressed the FGR-induced increases in A2m, Neuroserpin, CD200, and Ubb levels. (E and F) However, no significant improvements were observed in Ube1 or Otub1 expression changes with either treatment. (G) Representative images of Western blotting bands.

Given the observed improvements in brain dysfunctions associated with FGR following the MSC administration (Figure 5), we examined whether the expression levels of the six biomarker candidates in CSF were ameliorated by MSCs (Figures 7A–6G).

The FGR-induced an increase in A2m expression that persisted until PND 10. However, MSC administration on PND 4 significantly suppressed this increase by day 10 (Figure 7A). Similarly, the long-term upregulation of Neuroserpin in the FGR group was significantly reduced by MSC administration starting from the day 5 (Figure 7B). The FGR-induced increase in CD200 expression was also suppressed by MSC, particularly on day 14 (Figure 7C). The upregulation of Ubb caused by FGR was significantly reduced by MSC on days 5 and 7 (Figure 7D). In contrast to these four molecules, the FGR-induced expression changes in Ube1 and Otub1 were not particularly improved by the MSC administration (Figures 7E and 7F).

Overall, the behavioral experiments suggested that MSC administration could improve brain dysfunction caused by FGR (Figure 6). Consistent with this observation, MSCs alleviated FGR-induced changes in these biomarkers, showing overall improvement of Neuroserpin and partial improvement of A2m, CD200, and Ubb. These findings indicate that these four molecules may serve as valuable biomarkers and surrogate markers for both FGR-related brain dysfunction and treatment efficacy. Notably, the evaluation of Neuroserpin and Ubb expression enabled earlier detection of treatment responsiveness than the earliest observable improvements in behavioral tests, such as primitive reflex abilities, which became apparent around PND 10 following MSC administration (Figures 6A and 6B). While the MSC-mediated improvement in Ubb expression was no longer detectable after PND 10, Neuroserpin consistently reflected MSC-mediated therapeutic effects from PNDs 5 to 14.

### Serum Neuroserpin, α-2-macroglobulin, OX-2 membrane glycoprotein, and polyubiquitin-B provide a low-invasive diagnostic option

**Figure 8:**
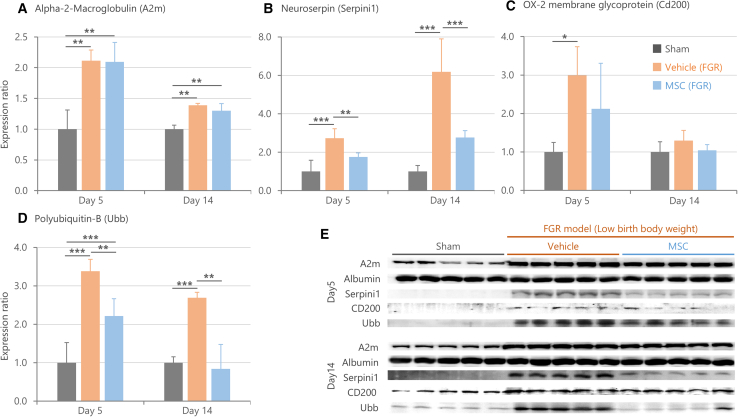
Serum expression levels and treatment responsiveness of candidate biomarker molecules (A–D) Relative expression levels of each molecule on postnatal days 5 and 14, with each time point normalized to the Sham group set to 1. The relative expression levels were standardized using albumin as an endogenous control. Data are presented as mean ± SD (*n* = 10/group/day biological replicates, ∗∗∗*p* < 0.001, ∗∗*p* < 0.01, ∗*p* < 0.05 by Steel-Dwass test). Serum expression levels of (A) Alpha-2-Macroglobulin (A2m), (B) Neuroserpin (Serpini1), (C) OX-2 membrane glycoprotein (CD200), and (D) Polyubiquitin-B (Ubb) were significantly elevated in the Vehicle group (FGR) compared to the Sham group on both postnatal days 5 and 14. Administration of mesenchymal stem/stromal cells (MSCs) on postnatal day 4 significantly suppressed the FGR-induced increases in Neuroserpin, and Ubb levels on days 5 and 14 (*n* = 10/group/day biological replicates, ∗∗∗*p* < 0.001, ∗∗*p* < 0.01, ∗*p* < 0.05 by Steel-Dwass test). (E) Representative images of Western blotting bands.

FGR infants are at high risk of being born with low birth weight and preterm. Consequently, collecting CSF from these infants for diagnosis purpose is highly invasive and impractical. Hence, we investigated whether the identified biomarker candidates could be detected in serum, considering potential leakage of CSF molecules into the bloodstream, and evaluated their abundance (Figure 8).

The results showed statistically significant increases in the serum levels of A2m, Neuroserpin, CD200, and Ubb in the FGR group compared to the Sham group (Figures 8A–8E). Notably, in the FGR group, Neuroserpin was the only molecule whose expression level was higher on Day 14 than at Day 5 (Figure 8B). Moreover, FGR-induced overexpression of Neuroserpin and Ubb were significantly suppressed by MSC (Figures 8B and 8D).

The results showed that the increased serum levels of A2m, Neuroserpin, CD200, and Ubb in the FGR group reflected similar expression changes as in the CSF (Figure 7). These molecules seemed to show correlations in their serum quantities with brain function, similarly to CSF. Therefore, these findings suggest that A2m, Neuroserpin, CD200, and Ubb may be valuable serum biomarkers for monitoring FGR-related brain dysfunction. In particular, the persistent elevation of Neuroserpin and Ubb in serum suggests it could serve as a stable biomarker, reflecting brain condition over time.

## Discussion

### Overview of key findings

This study is the first to comprehensively analyze CSF proteins collected from neonatal rats in a model of intrauterine hypoperfusion, identifying four biomarkers (Neuroserpin, A2m, CD200, and Ubb) whose expression correlates with birth weight and changes significantly in FGR conditions. We further demonstrated consistent alterations in brain tissue and serum, reinforcing their potential as early diagnostic predictors of FGR-induced brain developmental abnormalities. Given that 25–50% of infants with FGR experience neurodevelopmental delays by age two (29, 30), early detection is crucial for implementing timely interventions during the neonatal period of high neuroplasticity. Our results suggest that these biomarkers, especially Neuroserpin, could serve as practical indicators for identifying affected infants sooner, potentially guiding therapeutic decisions and enhancing outcomes.

### Significance of mild fetal growth restriction model

One of the key aspects of the present study is the utilization of the latest FGR model in neonatal rats, designed to induce a gradual and sustained restriction of blood flow to the fetus during the final quarter of gestation. In clinical practice, monitoring fetal blood flow is crucial for decisions on pregnancy management.^30^ When discussing research using FGR model animals, it is imperative to consider how these models are created and could correspond to human conditions. Our FGR model can generate a milder FGR fetus compared to already existing models,^31^^,^^32^^,^^33^ more closely mirroring the clinical spectrum of FGR.^16^^,^^17^ In typical cases of FGR, the blood flow decreases gradually, with abrupt reduction being rare.^34^ Traditional methods such as ligating uterine arteries^31^ and using clamps^32^ often result in the complete blood flow obstruction, biasing the selection of only severe FGR cases.^33^ In contrast, our model’s approach of gradual and chronic reduction of uterine blood flow more accurately simulates mild FGR conditions frequently encountered in clinical settings.^16^ Therefore, this realistic modeling underlines the translational potential of the identified biomarkers, which could be useful for the early detection of neurological outcomes in mild FGR cases, currently challenging to diagnose with existing technologies.

### Behavioral phenotypes and biomarker correlations

In our mild FGR model, established with a minimally invasive approach, we observed diminished primitive reflexes, impaired motor coordination, and deficits in spatial learning and memory—consistent with prior findings that lower birth weight correlated with more pronounced functional impairments.^16^ These pathological features mirror clinical observations in FGR infants.^18^^,^^19^ Similarly, models using relatively low-invasive coils to induce intrauterine hypoperfusion in rats revealed changes in both brain tissue and behavior, including reduced birth weight, decreased brain weight, ventricular enlargement, diminished cortical and white matter areas, hyperexcitability in spinal and cortical networks, somatosensory cortex disorganization, decreased primitive reflexes, excessive spontaneous movement, mild gait/posture deficits, hyperactivity, and social/memory disturbances.^35^^,^^36^^,^^37^ A more severe model using unilateral ligation further reported white matter damage, locomotor impairments, spasticity, disorganized somatosensory maps, spontaneous hyperactivity, and short-term memory deficits.^38^ Notably, among pups subjected to the same intrauterine hypoperfusion, those with lower birth weights exhibited these brain deficits more frequently, and the identified biomarkers significantly correlated with low birth weight. The four biomarker candidates—Neuroserpin, A2m, CD200, and Ubb—thus appear to reflect the behavioral anomalies stemming from abnormal brain function. Changes in their CSF and serum expression may indicate the severity of functional impairment, suggesting that they could serve as early predictive biomarkers of brain dysfunction. Although the exact mechanistic links between each biomarker and specific neural pathways require further investigation, these findings suggest that, collectively, the biomarkers capture ongoing neuropathological processes in FGR.

### Potential surrogate markers for therapeutic efficacy

An additional translational highlight is the possibility that these biomarker candidates also serve as surrogate markers for therapeutic monitoring. While no established treatments exist to reverse FGR-induced brain injuries, previous research has reported symptom alleviation through MSC administration. Research using the same FGR model as this study has demonstrated that umbilical cord-derived MSCs (UC-MSCs) can lead to long-term improvements in neurological maturation delays and abnormal brain function associated with FGR.^16^ Additionally, in models where intrauterine hypoperfusion was induced using coils, the intravenous administration of UC-MSCs mitigated spinal hyperexcitability and neurodevelopmental delays.^37^ Preclinical studies in animal models of developmental brain injury, such as hypoxic-ischemic conditions,^16^^,^^39^^,^^40^^,^^41^ stroke,^42^ neuroinflammation,^43^^,^^44^^,^^45^ and intraventricular hemorrhage,^46^ have further verified the neuroprotective effects of MSC administration. These benefits include enhancements in nutrient factors and extracellular vesicle secretion,^44^^,^^45^^,^^46^ brain metabolite profiles,^47^ energy metabolism,^41^ oligodendrocyte numbers and myelination,^41^^,^^46^ reactive gliosis,^46^^,^^48^ and reduction in cell death.^41^^,^^46^ Based on these findings, we hypothesized that MSCs could aid in repairing brain injuries associated with FGR. Hence, we evaluated the changes in the expression of biomarker candidates upon the MSC administration in the FGR model. The results revealed behavioral improvements indicative of alleviated brain function abnormalities post administration of MSCs (Figure 6), along with positive changes in the expression of each biomarker candidate in CSF and serum (Figures 7 and 8). Linking these findings suggests a possibility that these biomarker candidates could monitor therapeutic response, thereby reinforcing their potential utility as surrogate markers for reflecting therapeutic efficacy. Establishing surrogate markers to gauge brain function recovery would be a critical step in bringing novel therapies closer to clinical use and guide more personalized intervention strategies.

### Neuroserpin as a leading candidate

Among the identified biomarker candidates, Neuroserpin stands out as the most promising for predicting brain developmental abnormalities associated with FGR, owing to its brain-specific expression^49^ and consistent changes in both CSF and serum. While collecting CSF from FGR infants with low birth weight is impractical and potentially dangerous, blood testing remains the easiest, safest, and most practical alternative. However, FGR induces systemic pathological changes beyond the brain, causing blood biomarkers to reflect abnormalities in multiple organs, which complicate brain-specific diagnoses. To address this issue, it is ideal to use biomolecules specifically expressed in the brain that can leak into the bloodstream, providing a more accurate representation of brain conditions. Neuroserpin meets these criteria, as it is locally synthesized in brain neurons, making it less susceptible to systemic or non-neural influences. Furthermore, Neuroserpin exhibits stable and consistent expression changes. Therefore, Neuroserpin is a strong and promising candidate as a blood-based biomarker for the early detection of FGR-induced brain developmental abnormalities.

Neuroserpin is a serine protease inhibitor that regulates tissue plasminogen activator (tPA) in the brain.^50^ While tPA primarily initiates the fibrinolytic system, it also plays non-coagulant roles in the brain, such as regulating neurovascular unit permeability and synaptic plasticity during brain development and repair processes, promoting brain tissue reorganization.^51^ However, excessive tPA activity leads to cell death and tissue injury due to its neurotoxicity, necessitating strict regulation.^25^ Neuroserpin selectively inhibits excessive tPA activity, exerting neuroprotective functions.^25^ Indeed, its genetic deficiency causes abnormalities in neural circuit formation and synaptic plasticity, ultimately increasing the risk of emotional regulation disorders.^52^ Therefore, Neuroserpin is hypothesized to promote normal brain development by protecting against disrupted neural circuit formation caused by environmental stimuli during development.

During fetal and neonatal periods, Neuroserpin is strongly expressed in cortical regions associated with learning, memory, and behavior.^53^^,^^54^ Its expression is particularly prominent in subplate neurons, which regulate synaptogenesis and dynamics of newborn neurons in the cerebral cortex.^55^ Neuroserpin may be secreted from subplate neurons and involved in endoplasmic reticulum stress within these cells.^56^ Neuroserpin plays a neuroprotective role and promotes normal brain development when neural circuit formation is disrupted during development.^57^ However, its specific role and function in the developing brain under FGR conditions remain unclear. Investigating Neuroserpin’s role in the injured brain during the developmental stage is crucial for understanding the pathophysiology and mechanisms underlying NDDs related to FGR, paving the way for novel diagnostic and therapeutic strategies.

### Other biomarker candidates: α-2-macroglobulin, OX-2 membrane glycoprotein, and polyubiquitin-B

A2m is an acute-phase protein regulating protease activity during inflammation, preventing tissue damage.^26^ Predominantly expressed in astrocytes, A2m is upregulated during neural injury, aiding repair^58^ and present in fetal astrocytes, indicating responsiveness to intrauterine hypoperfusion.^59^ We observed an increased A2m in astrocytes with elevated levels in the FGR group until PND 7, decreasing by day 10, suggesting post-birth injury alleviation. Systemically synthesized A2m can affect brain development by entering the CSF and brain tissues,^26^^,^^60^^,^^61^ making it a potential marker for FGR-related brain development impacts. Differentiating brain-derived from other sources of A2m could enhance pathological understanding.

CD200 is a membrane glycoprotein regulating macrophages^27^ and primarily expressed in oligodendrocytes.^62^^,^^63^^,^^64^ It suppresses excessive microglial activation, which is increased in FGR models.^65^ CD200 upregulation may be triggered by IFN-γ and TNF-α during microglial activation.^66^ Elevated CD200 levels in the CSF may reflect heightened microglial activity, a common feature in ASD and ADHD,^67^^,^^68^^,^^69^ suggesting CD200 as a potential predictor for these disorders. Further research is needed to elucidate the relationship between increased CD200 in FGR and excessive microglial activation. Additionally, CD200 protects oligodendrocytes and myelinated nerve fibers from inflammation^70^ and exhibits dopaminergic neuroprotective functions.^62^^,^^71^ FGR-related intrauterine hypoperfusion is clinically associated with white matter damage, reduced cortical and hippocampal neurons, abnormal neural functions, decreased oligodendrocytes, and delayed myelination.^65^^,^^72^^,^^73^^,^^74^^,^^75^^,^^76^^,^^77^^,^^78^ Thus, increased CD200 expression in the FGR group may indicate an attempt to protect brain tissues from insufficient blood flow-induced damage.

Ubb is essential for intracellular protein degradation and metabolic turnover.^28^ In the brain, Ubb regulates neural plasticity and synapse formation.^79^^,^^80^ It is crucial during brain development, with disruptions leading to impaired neuronal morphology and synapse development, and linked to NDDs often associated with FGR.^81^^,^^82^^,^^83^^,^^84^ Restoring Ubb levels can reverse these abnormalities.^84^ Elevated Ubb in the CSF of our FGR model may counteract abnormal neural development caused by FGR.

### Conclusions

Taken together, our data suggest that mild FGR, often clinically overlooked or detected late, can be flagged by changes in four CSF- and serum-detectable proteins. In particular, Neuroserpin—due to its neuron-specific expression—is poised to become a sensitive blood-based indicator for FGR-induced brain dysfunction. These findings advance translational research by offering new biomarkers for early postnatal detection and intervention strategies, with the ultimate goal of improving neurological outcomes in infants affected by mild FGR. By facilitating timely, individualized treatments during a critical neuroplastic window, this approach could expand therapeutic options and enhance long-term outcomes, thereby addressing a pressing need in neonatal and perinatal medicine.

### Limitations of the study

When considering clinical applications, it is crucial to discuss which patient groups can benefit from this study’s findings. Some of the identified biomarker candidates showed sustained expression changes from PNDs 4 to 14 in association with FGR. During this developmental window, the rat brain corresponds to approximately human gestational weeks 28–32 through the late neonatal stage (around post-conceptual age of 44 weeks).^85^^,^^86^ Given that FGR infants are frequently delivered preterm before 32 weeks of gestation,^20^ the findings may contribute to the early predictive diagnosis of FGR-related brain abnormalities and clearer understanding of neurological disorders in term and preterm infants. Thus, the biomarkers may serve as clinically useful indicators for detecting abnormal brain development during a critical window of heightened neuroplasticity.

This implies a limitation: the inability to collect CSF from neonatal rats on PNDs 0 to 3, corresponding to gestational weeks 24–27 in humans, due to technical limitation such as blood contamination. This period is pivotal for establishing basic brain structure and the initial development of neural circuits.^87^ Developing sampling techniques with minimal blood contamination could unveil earlier mechanistic insights, particularly for extremely low birth weight infants at high risk.

In addition, rats are polytocous (bearing multiple offspring) and exhibit altricial (developmentally immature) characteristics at birth, which are physiologically and developmentally distinct from the single-birth, precocial (mature) pattern observed in humans. We selected the FGR rat model for this study because previous research has identified distinct neurodevelopmental impairments in these animals, both behaviorally and histologically, making them well-suited for exploring potential biomarkers associated with functional brain abnormalities. To properly interpret our findings, it is important to consider the fundamental differences in reproductive strategies and neonatal brain development between rats and humans. Moreover, to ensure the broader applicability of our results, further studies using animal models that more closely approximate human physiology, as well as clinical samples, will be essential.

Discussing the challenges of applying the identified biomarker candidates to clinical settings is beneficial. Although birth weight correlated with biomarker expression levels and behavioral deficits, we could not longitudinally track individuals into adulthood, limiting our ability to confirm whether these biomarkers directly predict long-term neurological outcomes. While lower birth weight was associated with greater biomarker alterations and more pronounced functional impairments, we could not directly evaluate how these changes influence ultimate neurodevelopmental trajectories. Ideally, biomarker levels would be directly linked to neurological outcomes post-development. Unfortunately, CSF collection required sacrificing neonatal rats, thus precluding subsequent behavioral testing and longitudinal correlation analysis. Also, the limited blood volume that could be safely collected from FGR neonatal rats constrained this aspect. Overcoming these limitations may require validation in larger animal models, including sheep, goats, or pigs.^33^^,^^88^ Such models allow repeated sampling and more direct correlations of biomarker expression with behavioral measures within the same individuals.

Ultimately, human-based studies will be essential for verifying whether shifts in these biomarkers truly precede and predict NDDs in FGR infants. To establish clinically practical cutoff values, it will be necessary to compare blood levels of candidate biomarkers, including Neuroserpin, between infants with and without FGR using human serum samples. This approach will allow for the calculation of sensitivity, specificity, and optimal diagnostic thresholds, which are essential for translating our findings into routine neonatal screening and prognostic assessments.

Moreover, although we identified Neuroserpin, A2m, CD200, and Ubb as brain-related biomarkers, we did not systematically investigate their expression in peripheral organs. In particular, A2m, CD200, and Ubb are reported to be expressed outside the brain, making it difficult to ascertain whether changes in their circulating levels originate from the brain or reflect alterations in peripheral tissues. Furthermore, while Neuroserpin is generally regarded as brain-specific, we cannot exclude the possibility of ectopic expression in other organs under FGR conditions. Future studies should examine these proteins across a broader range of tissues to validate their specificity for FGR-related neurodevelopmental changes. Such investigations would help identify the most suitable molecules for indicating brain dysfunction, thereby enhancing the reliability of minimally invasive diagnostic and therapeutic monitoring. Mechanistically, each biomarker likely participates in complex, interrelated pathways underlying brain injury and plasticity. Unraveling these mechanisms from a neuroscience perspective is imperative—not only to establish robust diagnostic criteria but also to guide targeted therapeutic development. Determining how each protein influences neural circuit formation, synaptic remodeling, or inflammatory processes could reveal novel intervention points, thereby advancing the translational potential of these findings.

## Resource availability

### Lead contact

Further information and requests for resources and reagents should be directed to and will be fulfilled by the lead contact, Dr. Atsuto Onoda (3b13624@alumni.tus.ac.jp).

### Materials availability

No unique reagents or materials were generated in this study. Further information or resources are available from the lead contact upon reasonable request.

### Data and code availability

Data: All data supporting the findings of this study are available within the main text and supplementary materials. All raw data, including raw proteomic data, have been deposited to Mendeley Data and are publicly available as of the date of publication. The DOI for the dataset is https://doi.org/10.17632/jcmk7jf38s.2, as listed in the key resources table.

Code: This article does not report original code.

Additional information: Any additional information required to reanalyze the data reported in this article is available from the lead contact upon request.

## Acknowledgments

We are grateful to Ms. Azusa Okamoto and Ms. Tomoko Yamaguchi for their expert technical assistance. We thank Kentaro Taki for operational support in LC/MS/MS in the laboratory of the Division for Medical Research Engineering, Nagoya University Graduate School of Medicine. This work was supported by Nagoya University Hospital Funding for Clinical Development 2018 (Y.S.), the 10.13039/501100001691Japan Society for the Promotion of Science Grant-in-Aid for Fund for the Promotion of Joint International Research (Fostering Joint International Research (B)) 21KK0176 (M.T.), JSPS Grant-in-Aid for Early-Career Scientists 19K17324 (A.O.) and 18K15668 (Y.K.), JSPS Grant-in-Aid for JSPS Research Fellows 18J01110 (A.O.), the 10.13039/100008732Uehara Memorial Foundation (A.O.), the Kawano Masanori Memorial Public Interest Incorporated Foundation for Promotion of Pediatric (A.O.), the Toyoaki Scholarship Foundation (A.O.), the 10.13039/501100003837Ichiro Kanehara Foundation for the Promotion of Medical Sciences and Medical Care (A.O.), and the 10.13039/100017778Public Foundation of Chubu Science and Technology Center (A.O.). The funders had no role in the preparation of the article or the decision to publish it.

## Author contributions

Conceptualization, A.O., Y.K., M.H., and Y.S.; methodology, A.O., Y.K., M.T., and Y.S.; investigation, A.O., Y.K., and K.U.; visualization, A.O., Y.K., and K.U.; funding acquisition, A.O., Y.K., M.T., and Y.S.; project administration, A.O., M.H., S.S., and Y.S.; supervision, J.O.C., S.S., M.H., and Y.S.; writing—original draft, A.O.; writing—review and editing, Y.K., J.O.C., K.U., S.S., M.T., M.H., and Y.S. All authors have read and approved the final article and agree to be accountable for all aspects of the work.

## Declaration of interests

A.O., Y.K., S.S., M.T., M.H., and Y.S. declare the following potential conflicts of interest with respect to the research, authorship, and/or publication of this article: A.O., Y.K., S.S., M.T., M.H., and Y.S. have a patent for the application of quantitative assessment index for fetal growth restriction. J.O.C. and K.U. declare that they have no competing interests.

## Declaration of generative AI and AI-assisted technologies

In preparing this article, we used ChatGPT (OpenAI, GPT o1 pro, accessed on February 24, 2025) to obtain suggestions for improving English expressions. In every case, we submitted the relevant text to ChatGPT with a prompt similar to: “Please proofread the following text for grammar, clarity, and style while preserving its scientific meaning.” All final decisions regarding text revisions were made by the authors, who assume full responsibility for the content of the article. In addition, we consulted the following references as guidelines on the academic use of generative AI and AI-assisted technologies during the writing and submission process.

(1) Nature Editorial. *Tools such as ChatGPT threaten transparent science; here are our ground rules for their use. Nature.* 2023; 613(7945):612. https://doi.org/10.1038/d41586-023-00191-1.
(2) Thorp HH. *ChatGPT is fun, but not an author. Science.* 2023; 379(6630):740. https://doi.org/10.1126/science.adg7879.

## STAR★Methods

### Key resources table

**Table:** 

REAGENT or RESOURCE	SOURCE	IDENTIFIER
**Antibodies**		

Rabbit anti-Albumin	Proteintech Inc. (IL, USA)	16475-1-AP; RRID:AB_2242567
Rabbit anti-A2m	Abcam (Cambridge, UK)	ab58703; RRID:AB_879541
Rabbit anti-CD200	Proteintech Inc.	14057-1-AP; RRID:AB_2878004
Rabbit anti-Ube1	Proteintech Inc.	15912-1-AP; RRID:AB_2211462
Rabbit anti-Otub1	Abcam	ab101471; RRID:AB_10858112
Rabbit anti-Neuroserpin	Abcam	ab33077; RRID:AB_956293
Rabbit anti-Ubb	StressMarq Biosciences Inc. (BC, Canada)	SPC-119; RRID:AB_2285248
HRP-conjugated goat anti-rabbit IgG	Rockland Immunochemicals Inc. (PA, USA)	611-603-122; RRID:AB_218606
Mouse anti-NeuN	Merck SA (Darmstadt, Germany)	MAB377; RRID:AB_2298772
Mouse anti-Olig2	Proteintech Inc.	66513-1-Ig; RRID:AB_2881876
Mouse anti-S100b	Abcam	ab4066; RRID:AB_304258
Mouse anti-Iba1	Santa Cruz Biotechnology (TX, USA)	sc-32725; RRID:AB_667733
Goat anti-mouse IgG (ATTO 550)	Rockland Immunochemicals Inc.	610-154-040; RRID:AB_2614862
Goat anti-rabbit IgG (ATTO 488)	Rockland Immunochemicals Inc.	611-152-122; RRID:AB_10893018

**Biological samples**		

Rat cerebrospinal fluid (CSF)	Obtained in this study’s animal experiments	Collected on postnatal days (PNDs) 4, 5, 7, 10, and 14
Rat serum	Same as above	Obtained by cardiac puncture and centrifugation
Rat brain tissue (fixed/frozen)	Same as above	Harvested on PND 10

**Chemicals, peptides, and recombinant proteins**		

EMEM Alpha modification	Sigma-Aldrich (St. Louis, MO)	M4526
Fetal bovine serum (FBS)	Hyclone (Logan, UT)	SH30910.03
Human fibroblast GF-2	Miltenyi Biotec (Bergisch Gladbach, Germany)	130-093-840
GlutaMAX I (2 mM)	Thermo Fisher Scientific Inc. (Waltham, MA)	35050061
Kanamycin sulfate (0.1 mg/mL)	Sigma-Aldrich (St. Louis, MO)	25389-94-0
Hank’s Balanced Salt Solution (HBSS)	Thermo Fisher Scientific Inc.	14170112
Protease inhibitor cocktail (Complete tablet, EDTA-free)	Roche Diagnostics (Basel, Switzerland)	4693116001
Ameroid constrictor (inner diameter 0.40 mm)	MSH Systems, Inc. (Tokyo, Japan)	SW-MICE-0.4-SS
Tandem Mass Tag™ (TMT) System (Sixplex)	Thermo Fisher Scientific Inc.	A44522
Immobilon Western Chemiluminescent HRP Substrate	Merck SA (Darmstadt, Germany)	41116010
ImmunoStar Zeta	FUJIFILM Wako Pure Chemical Corporation (Osaka, Japan)	295-72404
Tissue-Tek OCT compound	Sakura Finetek Japan Co., Ltd. (Tokyo, Japan)	4583
ProLong Diamond Antifade Reagent	Thermo Fisher Scientific Inc.	P36970

**Critical commercial assays**		

Pierce BCA Protein Assay kit	Thermo Fisher Scientific Inc.	A55864

**Deposited data**		

Proteomic raw data	Mendeley Data	https://doi.org/10.17632/jcmk7jf38s.2

**Experimental models: Cell lines**		

Human bone marrow–MSCs	Lonza (Basel, Switzerland)	PT-2501

**Experimental models: Organisms/strains**		

Sprague-Dawley rats	Japan SLC, Inc. (Shizuoka, Japan)	Approval numbers: 26128, 27191, 28002, 29015

**Software and algorithms**		

Scaffold	Proteome Software Inc. (Portland, OR)	version 4.4.8
Proteome Discoverer	Thermo Fisher Scientific Inc.	version 1.4
MASCOT	Matrix Science Inc. (Boston, MA)	version 2.6.0
Python	Python Software Foundation	version 3.11.5
Pandas	NumFOCUS	version 2.0.3
Matplotlib	NumFOCUS	version 3.7.1
SciPy	NumFOCUS	version 1.11.1
Image Lab software	Bio-Rad Laboratories (TX, USA)	version 6.1
Fiji/ImageJ	Open-source	version 2.13.1
ANY-maze™ Video Tracking System	Stoelting Co. (Wood Dale, IL)	10-000-284

**Other**		

Rat nesting material	Shepherd Specialty Papers, Inc. (TN, USA)	Shepherd Shack for Rat
Histo-Tek Hyfluid for Coolant used during OCT embedding	Sakura Finetek Japan Co., Ltd.	PINO-HF

### Experimental model and study participant details

All animal experiments were approved by the Nagoya University Animal Experiment Committee (Nagoya, Aichi Prefecture, Japan; Approval Numbers: 26128, 27191, 28002, 29015) and conducted in accordance with the Regulations on Animal Experiments at Nagoya University. All experiments were performed in a double-blind fashion and in accordance with the Animal Research: Reporting *In Vivo* Experiments (ARRIVE) guidelines for the care and use of laboratory animals.^89^ All sample collection was performed under isoflurane anesthesia, and all efforts were made to minimize the number of animals used and their suffering. The number of animals was kept to the minimum required to achieve statistical significance. Pregnant Sprague-Dawley rats were purchased from Japan SLC, Inc. (Shizuoka, Japan). All dams were housed individually in cages under controlled conditions (temperature: 22 ± 1°C, humidity: 50 ± 5%) with a 12-h light/12-h dark cycle and *ad libitum* access to food and water.

In the study design A (Figure 8), the dams were randomly assigned to Control (non-treated, *n* = 8), Sham (surgical procedure without hypoperfusion, *n* = 14), or Intrauterine hypoperfusion (ameroid constrictor-attached, *n* = 19) groups. Postnatally, thirty pups from Control, twenty-six from Sham, and twenty-seven from Intrauterine Hypoperfusion (FGR group) were used with low birth body weight below FGR threshold (FGR group). Control pups were used to calculate FGR model threshold, with their organs being used for another research project. From Sham and Intrauterine hypoperfusion groups, twenty pups each were used for proteomic analysis at PNDs 4 and 5 (*n* = 10/group/day), forty-five pups each for Western blotting at PNDs 4, 5, 7, 10, and 14 (*n* = 15/group), and ten pups each for histological analysis at PND 10 (*n* = 10/group).

In the study design B (Figure 8), dams were randomly assigned to Sham (*n* = 10) or Intrauterine hypoperfusion (*n* = 26) groups, with the latter split into vehicle-treated (*n* = 13) and MSC-treated (*n* = 13) subgroups. Postnatally, forty-six pups were allocated to Sham and 109 to Intrauterine hypoperfusion (61 vehicle-treated, 48 MSC-treated). Sixty-five pups from Sham, vehicle-treated non-FGR (vehicle A), vehicle-treated FGR (vehicle B), and MSC-treated FGR groups underwent behavioral tests at PNDs 8–11, 30–31, and 140–141 (n = 7–11/group). Also, ninety pups from Sham, vehicle-treated FGR, and MSC-treated FGR groups were used for Western blotting at PNDs 5, 7, 10, and 14 (*n* = 10–20/group). The minimal sample size was calculated to achieve 80% power of testing with an α error rate of 5%, based on preliminary experiments that assumed an effect size of 1.5 in the behavioral test, the primary endpoint.

### Method details

#### FGR model creation

The FGR model was created according to previously published methods.^16^ Intrauterine hypoperfusion surgery was performed on gestational day 17, corresponding to 20–25 weeks of human gestation,^90^ when FGR often occurs.^91^ After confirming that the flexor reflex was suppressed by 2.5% isoflurane anesthesia, the rat was placed in a supine position on a 37°C heated operating table. Abdomen of the rat was disinfected with alcohol, and body hair was removed with electric clippers, taking care not to damage the papillae. Under 2% isoflurane continuous anesthesia, a 1.5 cm incision was made in the middle of the abdominal skin and muscle layer, slightly above the height of the second papilla from the bottom. The uteri were then drawn out, and the fetuses were counted. One side of the uterus was returned to the abdominal cavity, while the other side was kept outside, covered with gauze, and humidified and warmed with 37°C physiological saline and a light bulb. The right and left ovarian and uterine arteries were sequentially dissected from the accompanying veins. A hydrophilic vessel constrictor with an inner diameter of 0.40 mm (Ameroid constrictor; SW-MICE-0.4-SS; MSH Systems, Inc.; Tokyo, Japan) was then attached on each of the four arteries (Figure 1A). The muscle and skin layers were sutured using continuous stitches, and the incision wound was closed. After suturing, the rat was placed in a supine position on the operating table for 10 min under 2% isoflurane anesthesia. In a previous study, we used a laser blood flowmeter ω zone (OMEGA WAVE, Inc., Tokyo, Japan) to measure uterine blood flow following ameroid constrictor placement and confirmed a significant and gradual reduction in uteroplacental perfusion.^16^ By the 20th day of pregnancy, the day before delivery, the vessel constrictor reduces blood flow to approximately 60% compared to the non-constrictor group. This reduction in uterine blood flow resulted in lower fetal weights, forming the basis for our FGR definition in this study. Because rats are polytocous, it is challenging to track which specific pups experienced the most reduced blood in blood flow after birth. Therefore, we adopted a threshold of 1.5 SD below the untreated group’s mean birth weight to define FGR pups, ensuring a consistent and practical criterion for our model. Regarding the Sham group, the same surgical procedure was performed as described above, except that the vessel constrictor was not attached. To minimize stress on the rats, nest material (Shepherd Shack for Rat; Shepherd Specialty Papers, Inc.; TN) as enrichment devices was placed in the cages on the 15th day of gestation, and every effort was made to avoid unnecessary stimulation.

#### Preparation and administration of mesenchymal stem/stromal cells (MSCs)

Human bone marrow–MSCs were purchased from Lonza (PT-2501, Basel, Switzerland) and cultured in EMEM Alpha modification (Sigma-Aldrich, St. Louis, MO) with 10% fetal bovine serum (SH30910.03, Hyclone, Logan, UT), 1 ng/mL human fibroblast GF-2 (130-093-840, Miltenyi Biotec, Bergisch Gladbach, Germany), 2 mM GlutaMAX I (Thermo Fisher Scientific Inc., Waltham, MA), and 0.1 mg/mL kanamycin sulfate at 37°C in 95% air and 5% carbon dioxide. Cells from passages 6 through 8 were used for experiments. On postnatal day (PND) 4, the right external jugular vein of the pups was exposed under 2% isoflurane anesthesia on a 37°C heating plate. Using a 35-gauge needle, MSCs (10^5^ cells/60 μL), or vehicle solution (Hank’s Balanced Salt Solution, Thermo Fisher Scientific Inc.) were injected via the right jugular vein.

#### Sample collection

Cerebrospinal fluid (CSF) was collected on PNDs 4, 5, 7, 10, and 14 using the transcutaneous cisterna magna puncture method with a microcapillary tube (outer diameter: 0.90 mm, inner diameter: 0.63 mm, 20 μL, Drummond Scientific Company, Broomall, PA), following the method described in previous studies.^92^ Blood was collected via cardiac puncture and placed into serum separation tubes (FG-SRMS; TAIYO COMPANY CO., Ltd, Osaka, Japan). The blood was centrifuged at 1,500 × g for 15 min at 4°C to obtain the serum. Both the collected CSF and serum were supplemented with a protease inhibitor cocktail (Complete tablet, EDTA-free, Roche Diagnostics, Basel, Switzerland) and stored at −80°C until further analysis. Total protein concentrations were quantified using the Pierce BCA Protein Assay kit (Thermo Fisher Scientific Inc.).

Brain tissues were collected on PND 10. Pups were anesthetized with isoflurane, then transcardially perfused with phosphate buffered saline (PBS) containing heparin, followed by 4% paraformaldehyde in PBS. The brains were collected, post-fixed in 4% paraformaldehyde in PBS for 24 h, and then cryoprotected in PBS-sucrose solutions with 0.1% sodium azide at incremental sucrose concentrations (10% for 10 h, 20% for 10 h, 30% for 30 h). The brains were then embedded in Tissue-Tek OCT compound (Sakura Finetek Japan Co., Ltd., Tokyo, Japan), immediately frozen in Histo-Tek Hyfluid (Sakura Finetek Japan Co., Ltd.) at −80°C, and stored at −80°C until further analysis.

#### Comprehensive analysis of proteins in CSF

The protein concentration in CSF at PNDs 4 and 5 of each pup were adjusted to 100 μg/200 μL and processed for trypsin digestion for 16 h at 37 °C following reduction reactions and alkylation reactions. The peptides were labeled with the Tandem Mass Tag system (Sixplex TMT, Thermo Fisher Scientific Inc.). Protein amounts were quantified using liquid chromatography/tandem mass spectrometry (LC/MS/MS) with an UltiMate3000 RSLCnano LC system (Dionex Co., Amsterdam, Netherlands) in combination with an Orbitrap Fusion mass spectrometer (Thermo Fisher Scientific Inc.). A nanocapillary column (150 mm × 75 μm i.d., Nikkyo Technos Co., Tokyo, Japan) via a nanoelectrospray ion source was used. Reversed-phase chromatography was performed with a linear gradient (0 min, 5% B; 100 min, 40% B) of solvent A (2% acetonitrile with 0.1% formic acid) and solvent B (95% acetonitrile with 0.1% formic acid) at a flow rate of 300 nL/min. A precursor ion scan was performed at a 400–1600 m/z range before tandem MS analysis. Tandem MS was conducted with quadrupole separation at 0.8 Th, HCD fragmentation at 30% normalized collision energy, and fast scan MS analysis. Only precursors with charge states 2–6 were sampled, with a dynamic exclusion time of 15 s and a tolerance of 10 ppm. The instrument operated in 3-s cycles.

Proteins were identified and validated using Scaffold software (version Scaffold_4.4.8, Proteome Software Inc., Portland, OR), with data analysis performed using Proteome Discoverer 1.4 (Thermo Fisher Scientific Inc.) and the MASCOT search engine (version 2.6.0, Matrix Science Inc., Boston, MA), referencing the UniProt protein database (release 2021_01). Raw proteome data were submitted to the Japan Proteome Standard Repository/Database (jPOST) with the accession number JPST003241 (PXD054372).^93^

To statistically analyze the protein profile, Spearman’s rank nonparametric correlation coefficient was calculated with the birth body weight of the pups, including both Sham (*n* = 10) and Intrauterine hypoperfusion (*n* = 10) groups. Half of the intrauterine hypoperfusion group was randomly selected for birth weights below the FGR criteria, and the other half was randomly selected for birth weights above the criteria. Based on the correlation coefficient, *p*-values and False Discovery Rate (Q-value of Storey’s method) were calculated. To identify proteins with low birth weight-dependent alterations, a threshold was set for a consistent trend of *p*-value <0.05 and Q-value <0.1 on both PNDs 4 and 5.

#### Quantification of biomarker candidates using western blotting

Proteins in the CSF and serum were mixed with denaturing sample buffer (125 mM Tris-HCl [pH 6.8], 20% glycerol, 4% w/v sodium dodecyl sulfate, 0.001% w/v bromophenol blue, and 10% mercaptoethanol) and denatured by heating for 5 min at 95°C. Each sample (20 μg per lane) was loaded onto either a 10% SDS-polyacrylamide gel (for Albumin, A2m, CD200, Ube1, and Otub1) or a 12% gel (for Neuroserpin and Ubb) and electrophoresed at 110 V for 30 min at room temperature, followed by 150 V for 110 min at 4°C. The separated proteins were then electroblotted onto a polyvinylidene difluoride membrane for 1 h at 400 mA at room temperature. After blocking with 5% skim milk in Tris-buffered saline (pH 7.4) containing 0.1% Tween 20 (TBS-T), the membranes were incubated for 15 h at 4°C with primary antibodies: rabbit anti-Albumin (16475-1-AP, Proteintech Inc., IL; 1:5000), rabbit anti-A2m (ab58703, Abcam, Cambridge, UK; 1:1000), rabbit anti-CD200 (14057-1-AP, Proteintech Inc.; 1:1000), rabbit anti-Ube1 (15912-1-AP, Proteintech Inc.; 1:1000), rabbit anti-Otub1 (ab101471, Abcam; 1:1000), rabbit anti-Neuroserpin (ab33077, Abcam; 1:1000), or rabbit anti-Ubb (SPC-119, StressMarq Biosciences, BC, Canada; 1:1000). This was followed by a 2-h incubation with secondary horseradish peroxidase (HRP)-conjugated anti-rabbit IgG (611-603-122, Rockland Immunochemicals, Inc., PA; 1:5000) at room temperature. Immunoblots were thoroughly washed with TBS-T between each step. Secondary antibody binding was visualized by chemiluminescence using Immobilon Western Chemiluminescent HRP Substrate (Merck SA, Darmstadt, Germany) for Albumin or ImmunoStar Zeta (FUJIFILM Wako Pure Chemical Corporation, Osaka, Japan). Detected signals were quantified by scanning images with a ChemiDoc MP System (Bio-Rad Laboratories, Inc., TX, USA) and analyzed using Image Lab software (Bio-Rad Laboratories). The band densities for Albumin (65 kDa), A2m (170 kDa), CD200 (45 kDa), Ube1 (120 kDa), Otub1 (35 kDa), Neuroserpin (50 kDa), and Ubb (35 kDa) were quantified with background subtraction, and their values were normalized to the corresponding Albumin value in each sample.

#### Behavioral tests

Two male rats per dam were randomly selected for behavioral tests. The rats were transferred to a room with 30 lux lighting 1 h before testing to habituate them to the room’s conditions. After each trial, the entire apparatus was cleaned with disinfectant ethanol and to remove any residual odor from previous rat and prevent olfactory-based bias.

At PNDs 8–11, the Negative geotaxis test was conducted to assess primitive reflex response. The pups were placed an anti-slip mat on a 30° slope, facing their head downward (Figure 5C). The time taken for the pups to rotate 180° and face upward was measured. Also, a clime-up score was assigned based on the rotation time: five points for 0–15 s, four points for 15–30 s, three points for 30–45 s, two points for 45–60 s, one point for over 60 s, and zero points for no reaction or falling.

At 30–31 and 140–141 days of age, the Rota-rod test was performed to evaluate sensorimotor coordination. The day before examination, rats underwent training at 4 rpm to acclimate them to the environment and the rods. When the actual examination was conducted, the rats were placed on rotating rods that accelerated from 4 to 40 rpm over 5 min (Figure 5F). The duration of stable walking on the rotarod was measured to assess their balance, coordination, and stamina. Each rat was tested twice a day, with a 4-h break between sessions, totaling four observations over 2 days.

Additionally, Y-maze test was conducted on days 30 and 140 of age to analyze short-term spatial working memory. Rats were initially placed at the center of the Y-maze and allowed to freely explore the three arms (Arm1, 2, and 3). The entry sequence was recorded over 8 min, and the entry pattern for effective observational behavior was assessed. Correct entry patterns were defined as entries into all three arms consecutively, such as 1-2-3, 1-3-2, 2-1-3, 2-3-1, 3-1-2, or 3-2-1 (Figure 5H). The number of three consecutive entries into different arms was divided by the total number of arm entries minus one and then multiplied by 100 to obtain a value that serves as an index of spatial working memory.

Open field test and Novel object recognition test were conducted on days 30 and 140 of age to assess spontaneous movement and recognition memory, respectively. Each rat was placed in the center of an open field (100 cm square) (see Figure S3T). The center of the open field was lit with approximately 100 lux to reduce the stress caused by intense lighting. Their behaviors were recorded for 5 min using an automated tracking system (ANY-mazeTM, video Tracking System; Stoelting Co., Wood Dale, IL). The analyzed behaviors included distance travel, mobile time, immobile time, mobile episodes, and immobile episodes in all areas, central area, and peripheral area. Additionally, line crossings in all areas and the number of entries into the central and peripheral areas were evaluated.

After the open field test, Novel object recognition test was performed using the same open field. The rats were habituated for three days, freely exploring the empty open field for 5 min each day. On the day following habituation, the rats spent 5 min in the open field with two familiar objects. After 24 h, the rats were placed in the open field with one familiar object and one novel object (see Figure S3U). The time spent by the rats around each object during a 5-min period was measured. The recognition index was calculated as the time spent with the new object divided by the sum of the time spent with the new object and the time spent with the familiar object.

#### Histological analysis by immunofluorescence

Brain sections with 10-μm thick were prepared from the frozen blocks using a Tissue-Tek Polar instrument (Sakura Finetek Japan Co., Ltd.) and mounted onto a glass slide. The sections were air-dried for 48 h to prevent moisture interference. The dried sections were submerged in PBS to remove the embedding compound and then blocked with 10% normal goat serum (IHR-8136, ImmunoBioScience, Corp., WA, USA) in PBS for 1 h at room temperature. The sections were incubated with primary antibodies—mouse anti-NeuN (MAB377, Merck SA; 1:200), mouse anti-Olig2 (66513-1-Ig, Proteintech Inc.; 1:100), mouse anti-S100b (ab4066, Abcam.; 1:200), or mouse anti-Iba1 (sc-32725, Santa Cruz Biotechnology, TX, USA; 1:200)—for 15 h at 4°C. After rinsing 3 times for 5 min each with PBS, the sections were incubated with secondary ATTO 550-conjugated goat anti-mouse IgG (610-154-040, Rockland Immunochemicals Inc.; 1:1000) for 3 h at room temperature. Following another set of rinses (3 times for 5 min each with PBS), the sections were incubated with primary antibodies—rabbit anti-CD200 (14057-1-AP, Proteintech Inc.; 1:100), rabbit anti-A2m (ab58703, Abcam; 1:100), rabbit anti-Ubb (SPC-119B, StressMarq Biosciences INC., BC, Canada; 1:100), or rabbit anti-Neuroserpin (ab33077, Abcam; 1:100)—for 15 h at 4°C. After rinsing 3 times for 5 min each with PBS, the sections were further incubated with secondary ATTO 488-conjugated goat anti-rabbit IgG (611-152-122, Rockland Immunochemicals Inc.; 1:500) for 3 h at room temperature. The sections were then rinsed 3 times for 5 min each with PBS and twice for 1 min each with distilled water. Nuclei were counterstained using Hoechst 33342 (346–07951, Dojindo Laboratories, Kumamoto, Japan). Prolong Diamond Antifade Reagent (P36970, Thermo Fisher Scientific Inc.) was used to prevent fading, and the slides were sealed.

Images for quantification were acquired using a confocal fluorescence microscope (LSM 900/LSM 900 Airyscan 2, ZEISS global, Jena, Germany). Identical microscope parameters were applied to all images. For each pup, 10 cells were randomly selected cells from the brain regions of the cerebral cortex, hippocampus, and thalamus for analysis (*n* = 10/group, totaling 100 cells in each group). The fluorescence intensities of A2m, CD200, Ubb, and Neuroserin in NeuN, S100b, Olig2, and Iba1 positive cells were quantified. Relative fluorescence intensity was calculated by setting the maximum value to 100 and the background to 1, and these values were graphed. Image analysis was performed using Fiji/ImageJ software, based on previous studies.^94^

### Quantification and statistical analysis

Birth body weight was presented using boxplots, showing the interquartile range with means indicated by cross marks (*n* = 26–30/group, biological replicate). Body weight gain (*n* = 26–27/group, biological replicate), biomarker candidate levels via western blotting (*n* = 10–20/group, biological replicate), and behavioral test results (n = 7–11/group, biological replicate) were represented as mean ± standard deviation (SD). Histological analysis results were depicted using bee-swarm and boxplots (*n* = 10/group, 100 cells/group). The Wilcoxon signed-rank test compared body weight gain between two groups. Other data were analyzed using the Steel-Dwass method. All statistical tests were two-tailed with significance set at *p*-value <0.05. Correlation between the protein expression (proteomics) and birth body weight were assessed using Spearman’s rank correlation coefficient, with *p*-values and Q-values (Storey’s method) computed (*n* = 10/group/day, biological replicate). Data analyses and visualization were conducted using Python (3.11.5) with Pandas (2.0.3), Matplotlib (3.7.1), and SciPy (1.11.1). No missing or modified data points were present.

## References

[1] NCHS. Percentage of children ages 5 to 17 years reported to have attention-deficit/hyperactivity disorder (ADHD), by sex, 1997-2021 (Indicator H6). NCfHS, 1997-2021.

[2] Zwaigenbaum L., Penner M. (2018). Autism spectrum disorder: advances in diagnosis and evaluation. BMJ.

[3] Levine T.A., Grunau R.E., McAuliffe F.M., Alderdice F.A. (2019). Early psychosocial development of small for gestational age and intrauterine growth-restricted children: a systematic review. J. Perinatol..

[4] Levine T.A., Grunau R.E., McAuliffe F.M., Pinnamaneni R., Foran A., Alderdice F.A. (2015). Early childhood neurodevelopment after intrauterine growth restriction: a systematic review. Pediatrics.

[5] Ruff C.A., Faulkner S.D., Rumajogee P., Beldick S., Foltz W., Corrigan J., Basilious A., Jiang S., Thiyagalingam S., Yager J.Y., Fehlings M.G. (2017). The extent of intrauterine growth restriction determines the severity of cerebral injury and neurobehavioural deficits in rodents. PLoS One.

[6] Wixey J.A., Chand K.K., Colditz P.B., Bjorkman S.T. (2017). Neuroinflammation in intrauterine growth restriction. Placenta.

[7] De Onis M., Blössner M., Villar J. (1998). Levels and patterns of intrauterine growth retardation in developing countries. Eur. J. Clin. Nutr..

[8] Lehner C., Harry A., Pelecanos A., Wilson L., Pink K., Sekar R. (2019). The feasibility of a clinical audit tool to investigate stillbirth in Australia–a single centre experience. Aust. N. Z. J. Obstet. Gynaecol..

[9] ACOG Practice Bulletin FGR (2021).

[10] Robinson J.S., Moore V.M., Owens J.A., McMillen I.C. (2000). Origins of fetal growth restriction. Eur. J. Obstet. Gynecol. Reprod. Biol..

[11] Meher S., Hernandez-Andrade E., Basheer S.N., Lees C. (2015). Impact of cerebral redistribution on neurodevelopmental outcome in small-for-gestational-age or growth-restricted babies: a systematic review. Ultrasound Obstet. Gynecol..

[12] Jang E.A., Longo L.D., Goyal R. (2015). Antenatal maternal hypoxia: criterion for fetal growth restriction in rodents. Front. Physiol..

[13] Geva R., Eshel R., Leitner Y., Valevski A.F., Harel S. (2006). Neuropsychological outcome of children with intrauterine growth restriction: a 9-year prospective study. Pediatrics.

[14] Malhotra A., Sepehrizadeh T., Dhollander T., Wright D., Castillo-Melendez M., Sutherland A.E., Pham Y., Ditchfield M., Polglase G.R., de Veer M. (2019). Advanced MRI analysis to detect white matter brain injury in growth restricted newborn lambs. Neuroimage Clin..

[15] Miller S.L., Huppi P.S., Mallard C. (2016). The consequences of fetal growth restriction on brain structure and neurodevelopmental outcome. J. Physiol..

[16] Kitase Y., Sato Y., Arai S., Onoda A., Ueda K., Go S., Mimatsu H., Jabary M., Suzuki T., Ito M. (2020). Establishment of a Novel Fetal Growth Restriction Model and Development of a Stem-Cell Therapy Using Umbilical Cord-Derived Mesenchymal Stromal Cells. Front. Cell. Neurosci..

[17] Herrera E.A., Alegría R., Farias M., Díaz-López F., Hernández C., Uauy R., Regnault T.R.H., Casanello P., Krause B.J. (2016). Assessment of in vivo fetal growth and placental vascular function in a novel intrauterine growth restriction model of progressive uterine artery occlusion in guinea pigs. J. Physiol..

[18] Samuelsen G.B., Pakkenberg B., Bogdanović N., Gundersen H.J.G., Larsen J.F., Graem N., Laursen H. (2007). Severe cell reduction in the future brain cortex in human growth–restricted fetuses and infants. Am. J. Obstet. Gynecol..

[19] Leitner Y., Fattal-Valevski A., Geva R., Bassan H., Posner E., Kutai M., Many A., Jaffa A.J., Harel S. (2000). Six-year follow-up of children with intrauterine growth retardation: long-term, prospective study. J. Child Neurol..

[20] Kingdom J., Ashwal E., Lausman A., Liauw J., Soliman N., Figueiro-Filho E., Nash C., Bujold E., Melamed N. (2023). Guideline No. 442: Fetal Growth Restriction: Screening, Diagnosis, and Management in Singleton Pregnancies. SOGC Clin. Pract. Guidel..

[21] Mimura K., Takagi K., Suzuki H., Iriyama T., Seki H. (2022). Diagnosis and management of fetal growth restriction and uteroplacental dysfunction in hypertensive disorders of pregnancy in Japan: a nationwide survey by the Japan Society for the Study of Hypertension in Pregnancy (JSSHP). Hypertens. Res. Pregnancy.

[22] Iliff J.J., Wang M., Liao Y., Plogg B.A., Peng W., Gundersen G.A., Benveniste H., Vates G.E., Deane R., Goldman S.A., Nagelhus E.A. (2012). A paravascular pathway facilitates CSF flow through the brain parenchyma and the clearance of interstitial solutes, including amyloid beta. Sci. Transl. Med..

[23] Olsson B., Zetterberg H., Hampel H., Blennow K. (2011). Biomarker-based dissection of neurodegenerative diseases. Prog. Neurobiol..

[24] Bivona G., Iemmolo M., Ghersi G. (2023). Cerebrospinal and Blood Biomarkers in Alzheimer's Disease: Did Mild Cognitive Impairment Definition Affect Their Clinical Usefulness?. Int. J. Mol. Sci..

[25] Kaur J., Zhao Z., Klein G.M., Lo E.H., Buchan A.M. (2004). The neurotoxicity of tissue plasminogen activator?. J. Cereb. Blood Flow Metab..

[26] Tiggelman A.M., Boers W., Moorman A.F., de Boer P.A., Van der Loos C.M., Rotmans J.P., Chamuleau R.A. (1996). Localization of α2-macroglobulin protein and messenger RNA in rat liver fibrosis: Evidence for the synthesis of α2-macroglobulin withinSchistosoma mansoniegg granulomas. Hepatology.

[27] Hoek R.M., Ruuls S.R., Murphy C.A., Wright G.J., Goddard R., Zurawski S.M., Blom B., Homola M.E., Streit W.J., Brown M.H. (2000). Down-regulation of the macrophage lineage through interaction with OX2 (CD200). Science.

[28] Alves-Rodrigues A., Gregori L., Figueiredo-Pereira M.E. (1998). Ubiquitin, cellular inclusions and their role in neurodegeneration. Trends Neurosci..

[29] Kitase Y., Sato Y., Ueda K., Suzuki T., Mikrogeorgiou A., Sugiyama Y., Matsubara K., Tsukagoshi Okabe Y., Shimizu S., Hirata H. (2020). A novel treatment with stem cells from human exfoliated deciduous teeth for hypoxic-ischemic encephalopathy in neonatal rats. Stem Cells Dev..

[30] Schwarze A., Gembruch U., Krapp M., Katalinic A., Germer U., Axt-Fliedner R. (2005). Qualitative venous Doppler flow waveform analysis in preterm intrauterine growth-restricted fetuses with ARED flow in the umbilical artery—correlation with short-term outcome. Ultrasound Obstet. Gynecol..

[31] Tashima L., Nakata M., Anno K., Sugino N., Kato H. (2001). Prenatal influence of ischemia-hypoxia-induced intrauterine growth retardation on brain development and behavioral activity in rats. Neonatology.

[32] Kazemi-Darabadi S., Akbari G. (2020). Evaluation of magnesium sulfate effects on fetus development in experimentally induced surgical fetal growth restriction in rat. J. Matern. Fetal Neonatal Med..

[33] Swanson A.M., David A.L. (2015). Animal models of fetal growth restriction: Considerations for translational medicine. Placenta.

[34] Lees C.C., Romero R., Stampalija T., Dall'Asta A., DeVore G.A., Prefumo F., Frusca T., Visser G.H.A., Hobbins J.C., Baschat A.A. (2022). The diagnosis and management of suspected fetal growth restriction: an evidence-based approach. Am. J. Obstet. Gynecol..

[35] Ohshima M., Coq J.-O., Otani K., Hattori Y., Ogawa Y., Sato Y., Harada-Shiba M., Ihara M., Tsuji M. (2016). Mild intrauterine hypoperfusion reproduces neurodevelopmental disorders observed in prematurity. Sci. Rep..

[36] Coq J.-O., Delcour M., Ogawa Y., Peyronnet J., Castets F., Turle-Lorenzo N., Montel V., Bodineau L., Cardot P., Brocard C. (2018). Mild intrauterine hypoperfusion leads to lumbar and cortical hyperexcitability, spasticity, and muscle dysfunctions in rats: implications for prematurity. Front. Neurol..

[37] Tsuji M., Mukai T., Sato Y., Azuma Y., Yamamoto S., Cayetanot F., Bodineau L., Onoda A., Nagamura-Inoue T., Coq J.O. (2023). Umbilical cord-derived mesenchymal stromal cell therapy to prevent the development of neurodevelopmental disorders related to low birth weight. Sci. Rep..

[38] Delcour M., Olivier P., Chambon C., Pansiot J., Russier M., Liberge M., Xin D., Gestreau C., Alescio-Lautier B., Gressens P. (2012). Neuroanatomical, sensorimotor and cognitive deficits in adult rats with white matter injury following prenatal ischemia. Brain Pathol..

[39] Zhu L.-H., Bai X., Zhang N., Wang S.-Y., Li W., Jiang L. (2014). Improvement of human umbilical cord mesenchymal stem cell transplantation on glial cell and behavioral function in a neonatal model of periventricular white matter damage. Brain Res..

[40] Sugiyama Y., Sato Y., Kitase Y., Suzuki T., Kondo T., Mikrogeorgiou A., Horinouchi A., Maruyama S., Shimoyama Y., Tsuji M. (2018). Intravenous administration of bone marrow-derived mesenchymal stem cell, but not adipose tissue-derived stem cell, ameliorated the neonatal hypoxic-ischemic brain injury by changing cerebral inflammatory state in rat. Front. Neurol..

[41] Robertson N.J., Meehan C., Martinello K.A., Avdic-Belltheus A., Boggini T., Mutshiya T., Lingam I., Yang Q., Sokolska M., Charalambous X. (2021). Human umbilical cord mesenchymal stromal cells as an adjunct therapy with therapeutic hypothermia in a piglet model of perinatal asphyxia. Cytotherapy.

[42] Tanaka E., Ogawa Y., Mukai T., Sato Y., Hamazaki T., Nagamura-Inoue T., Harada-Shiba M., Shintaku H., Tsuji M. (2018). Dose-dependent effect of intravenous administration of human umbilical cord-derived mesenchymal stem cells in neonatal stroke mice. Front. Neurol..

[43] Morioka C., Komaki M., Taki A., Honda I., Yokoyama N., Iwasaki K., Iseki S., Morio T., Morita I. (2017). Neuroprotective effects of human umbilical cord-derived mesenchymal stem cells on periventricular leukomalacia-like brain injury in neonatal rats. Inflamm. Regen..

[44] Thomi G., Surbek D., Haesler V., Joerger-Messerli M., Schoeberlein A. (2019). Exosomes derived from umbilical cord mesenchymal stem cells reduce microglia-mediated neuroinflammation in perinatal brain injury. Stem Cell Res. Ther..

[45] Thomi G., Joerger-Messerli M., Haesler V., Muri L., Surbek D., Schoeberlein A. (2019). Intranasally administered exosomes from umbilical cord stem cells have preventive neuroprotective effects and contribute to functional recovery after perinatal brain injury. Cells.

[46] Mukai T., Mori Y., Shimazu T., Takahashi A., Tsunoda H., Yamaguchi S., Kiryu S., Tojo A., Nagamura-Inoue T. (2017). Intravenous injection of umbilical cord-derived mesenchymal stromal cells attenuates reactive gliosis and hypomyelination in a neonatal intraventricular hemorrhage model. Neuroscience.

[47] Tanaka E., Ogawa Y., Fujii R., Shimonaka T., Sato Y., Hamazaki T., Nagamura-Inoue T., Shintaku H., Tsuji M. (2020). Metabolomic analysis and mass spectrometry imaging after neonatal stroke and cell therapies in mouse brains. Sci. Rep..

[48] Paton M.C.B., Allison B.J., Fahey M.C., Li J., Sutherland A.E., Pham Y., Nitsos I., Bischof R.J., Moss T.J., Polglase G.R. (2019). Umbilical cord blood versus mesenchymal stem cells for inflammation-induced preterm brain injury in fetal sheep. Pediatr. Res..

[49] Lebeurrier N., Liot G., Lopez-Atalaya J.P., Orset C., Fernandez-Monreal M., Sonderegger P., Ali C., Vivien D. (2005). The brain-specific tissue-type plasminogen activator inhibitor, neuroserpin, protects neurons against excitotoxicity both in vitro and in vivo. Mol. Cell. Neurosci..

[50] Osterwalder T., Contartese J., Stoeckli E.T., Kuhn T.B., Sonderegger P. (1996). Neuroserpin, an axonally secreted serine protease inhibitor. EMBO J..

[51] Yepes M., Lawrence D.A. (2004). Tissue-type plasminogen activator and neuroserpin: a well-balanced act in the nervous system?. Trends Cardiovasc. Med..

[52] Madani R., Kozlov S., Akhmedov A., Cinelli P., Kinter J., Lipp H.P., Sonderegger P., Wolfer D.P. (2003). Impaired explorative behavior and neophobia in genetically modified mice lacking or overexpressing the extracellular serine protease inhibitor neuroserpin. Mol. Cell. Neurosci..

[53] Kement D., Reumann R., Schostak K., Voß H., Douceau S., Dottermusch M., Schweizer M., Schlüter H., Vivien D., Glatzel M., Galliciotti G. (2021). Neuroserpin is strongly expressed in the developing and adult mouse neocortex but its absence does not perturb cortical lamination and synaptic proteome. Front. Neuroanat..

[54] Miranda E., Lomas D.A. (2006). Neuroserpin: a serpin to think about. Cell. Mol. Life Sci..

[55] Adorjan I., Tyler T., Bhaduri A., Demharter S., Finszter C.K., Bako M., Sebok O.M., Nowakowski T.J., Khodosevich K., Møllgård K. (2019). Neuroserpin expression during human brain development and in adult brain revealed by immunohistochemistry and single cell RNA sequencing. J. Anat..

[56] Kondo S., Al-Hasani H., Hoerder-Suabedissen A., Wang W.Z., Molnár Z. (2015). Secretory function in subplate neurons during cortical development. Front. Neurosci..

[57] Millar L.J., Shi L., Hoerder-Suabedissen A., Molnár Z. (2017). Neonatal hypoxia ischaemia: mechanisms, models, and therapeutic challenges. Front. Cell. Neurosci..

[58] Higuchi M., Ito T., Imai Y., Iwaki T., Hattori M., Kohsaka S., Niho Y., Sakaki Y. (1994). Expression of the alpha 2-macroglobulin-encoding gene in rat brain and cultured astrocytes. Gene.

[59] Lauro G.M., Fabrizi C., Businaro R., Fumagalli L., Torelli S., Gremo F. (1992). Human astroglial but not microglial cells synthesize α 2 in vitroin vitro. Ital. J. Neurol. Sci..

[60] Hayashida K., Tsuchiya Y., Kurokawa S., Hattori M., Ishibashi H., Okubo H., Sakaki Y. (1986). Expression of rat alpha 2-macroglobulin gene during pregnancy. J. Biochem..

[61] Møllgård K., Dziegielewska K.M., Saunders N.R., Zakut H., Soreq H. (1988). Synthesis and localization of plasma proteins in the developing human brain: integrity of the fetal blood-brain barrier to endogenous proteins of hepatic origin. Dev. Biol..

[62] Chamera K., Trojan E., Szuster-Głuszczak M., Basta-Kaim A. (2020). The potential role of dysfunctions in neuron-microglia communication in the pathogenesis of brain disorders. Curr. Neuropharmacol..

[63] Lyons A., Downer E.J., Crotty S., Nolan Y.M., Mills K.H.G., Lynch M.A. (2007). CD200 ligand–receptor interaction modulates microglial activation in vivo and in vitro: a role for IL-4. J. Neurosci..

[64] Zeis T., Enz L., Schaeren-Wiemers N. (2016). The immunomodulatory oligodendrocyte. Brain Res..

[65] Olivier P., Baud O., Evrard P., Gressens P., Verney C. (2005). Prenatal ischemia and white matter damage in rats. J. Neuropathol. Exp. Neurol..

[66] Chen Z., Marsden P.A., Gorczynski R.M. (2009). Role of a distal enhancer in the transcriptional responsiveness of the human CD200 gene to interferon-γ and tumor necrosis factor-α. Mol. Immunol..

[67] Verlaet A.A.J., Maasakkers C.M., Hermans N., Savelkoul H.F.J. (2018). Rationale for dietary antioxidant treatment of ADHD. Nutrients.

[68] Suzuki K., Sugihara G., Ouchi Y., Nakamura K., Futatsubashi M., Takebayashi K., Yoshihara Y., Omata K., Matsumoto K., Tsuchiya K.J. (2013). Microglial activation in young adults with autism spectrum disorder. JAMA Psychiatry.

[69] Rodriguez J.I., Kern J.K. (2011). Evidence of microglial activation in autism and its possible role in brain underconnectivity. Neuron Glia Biol..

[70] Koning N., Bö L., Hoek R.M., Huitinga I. (2007). Downregulation of macrophage inhibitory molecules in multiple sclerosis lesions. Ann. Neurol..

[71] Varnum M.M., Kiyota T., Ingraham K.L., Ikezu S., Ikezu T. (2015). The anti-inflammatory glycoprotein, CD200, restores neurogenesis and enhances amyloid phagocytosis in a mouse model of Alzheimer's disease. Neurobiol. Aging.

[72] Rees S., Bocking A.D., Harding R. (1988). Structure of the fetal sheep brain in experimental growth retardation. J. Dev. Physiol..

[73] Lodygensky G.A., Seghier M.L., Warfield S.K., Tolsa C.B., Sizonenko S., Lazeyras F., Hüppi P.S. (2008). Intrauterine growth restriction affects the preterm infant's hippocampus. Pediatr. Res..

[74] Teitelbaum P., Teitelbaum O., Nye J., Fryman J., Maurer R.G. (1998). Movement analysis in infancy may be useful for early diagnosis of autism. Proc. Natl. Acad. Sci. USA.

[75] Jansiewicz E.M., Goldberg M.C., Newschaffer C.J., Denckla M.B., Landa R., Mostofsky S.H. (2006). Motor signs distinguish children with high functioning autism and Asperger’s syndrome from controls. J. Autism Dev. Disord..

[76] Reid M.V., Murray K.A., Marsh E.D., Golden J.A., Simmons R.A., Grinspan J.B. (2012). Delayed myelination in an intrauterine growth retardation model is mediated by oxidative stress upregulating bone morphogenetic protein 4. J. Neuropathol. Exp. Neurol..

[77] Fung C., Ke X., Brown A.S., Yu X., McKnight R.A., Lane R.H. (2012). Uteroplacental insufficiency alters rat hippocampal cellular phenotype in conjunction with ErbB receptor expression. Pediatr. Res..

[78] Basilious A., Yager J., Fehlings M.G. (2015). Neurological outcomes of animal models of uterine artery ligation and relevance to human intrauterine growth restriction: a systematic review. Dev. Med. Child Neurol..

[79] Ambrozkiewicz M.C., Schwark M., Kishimoto-Suga M., Borisova E., Hori K., Salazar-Lázaro A., Rusanova A., Altas B., Piepkorn L., Bessa P. (2018). Polarity acquisition in cortical neurons is driven by synergistic action of Sox9-regulated Wwp1 and Wwp2 E3 ubiquitin ligases and intronic miR-140. Neuron.

[80] Hsia H.-E., Kumar R., Luca R., Takeda M., Courchet J., Nakashima J., Wu S., Goebbels S., An W., Eickholt B.J. (2014). Ubiquitin E3 ligase Nedd4-1 acts as a downstream target of PI3K/PTEN-mTORC1 signaling to promote neurite growth. Proc. Natl. Acad. Sci. USA.

[81] Park C.-W., Jung B.-K., Ryu K.-Y. (2020). Disruption of the polyubiquitin gene Ubb reduces the self-renewal capacity of neural stem cells. Biochem. Biophys. Res. Commun..

[82] Jung B.-K., Park C.-W., Ryu K.-Y. (2018). Temporal downregulation of the polyubiquitin gene Ubb affects neuronal differentiation, but not maturation, in cells cultured in vitro. Sci. Rep..

[83] Ambrozkiewicz M.C., Cuthill K.J., Harnett D., Kawabe H., Tarabykin V. (2020). Molecular evolution, neurodevelopmental roles and clinical significance of HECT-type UBE3 E3 ubiquitin ligases. Cells.

[84] Ryu H.-W., Park C.-W., Ryu K.-Y. (2014). Restoration of cellular ubiquitin reverses impairments in neuronal development caused by disruption of the polyubiquitin gene Ubb. Biochem. Biophys. Res. Commun..

[85] Semple B.D., Blomgren K., Gimlin K., Ferriero D.M., Noble-Haeusslein L.J. (2013). Brain development in rodents and humans: Identifying benchmarks of maturation and vulnerability to injury across species. Prog. Neurobiol..

[86] Clancy B., Finlay B.L., Darlington R.B., Anand K.J.S. (2007). Extrapolating brain development from experimental species to humans. Neurotoxicology.

[87] Workman A.D., Charvet C.J., Clancy B., Darlington R.B., Finlay B.L. (2013). Modeling transformations of neurodevelopmental sequences across mammalian species. J. Neurosci..

[88] Regnault T.R.H. (2003). Ruminant models of prenatal growth restriction. Reproduction.

[89] Kilkenny C., Browne W., Cuthill I.C., Emerson M., Altman D.G., NC3Rs Reporting Guidelines Working Group (2010). Animal research: reporting in vivo experiments: the ARRIVE guidelines. Br. J. Pharmacol..

[90] Salmaso N., Jablonska B., Scafidi J., Vaccarino F.M., Gallo V. (2014). Neurobiology of premature brain injury. Nat. Neurosci..

[91] Li X., Zhang W., Lin J., Liu H., Yang Z., Teng Y., Huang J., Peng Q., Lin X., Zhang J. (2021). Hypertensive disorders of pregnancy and risks of adverse pregnancy outcomes: a retrospective cohort study of 2368 patients. J. Hum. Hypertens..

[92] GBD 2013 Risk Factors Collaborators, Forouzanfar M.H., Alexander L., Anderson H.R., Bachman V.F., Biryukov S., Brauer M., Burnett R., Casey D., Coates M.M. (2015). Global, regional, and national comparative risk assessment of 79 behavioural, environmental and occupational, and metabolic risks or clusters of risks in 188 countries, 1990–2013: a systematic analysis for the Global Burden of Disease Study 2013. Lancet.

[93] Okuda S., Watanabe Y., Moriya Y., Kawano S., Yamamoto T., Matsumoto M., Takami T., Kobayashi D., Araki N., Yoshizawa A.C. (2017). jPOSTrepo: an international standard data repository for proteomes. Nucleic Acids Res..

[94] Schindelin J., Arganda-Carreras I., Frise E., Kaynig V., Longair M., Pietzsch T., Preibisch S., Rueden C., Saalfeld S., Schmid B. (2012). Fiji: an open-source platform for biological-image analysis. Nat. Methods.

