# Supplementary material for: Biomarkers for early detection and monitoring of abnormal brain development in mild fetal growth restriction
Source: iScience. 2025 Jul 30;28(9):113237. doi: 10.1016/j.isci.2025.113237 (PMC12392332; doi:10.1016/j.isci.2025.113237)
Supplement: Document S1. Figures S1–S3 [file mmc1.pdf]

iScience, Volume *28*

Supplemental information

Biomarkers for early detection and monitoring of abnormal brain development in mild fetal growth restriction

Atsuto Onoda, Yuma Kitase, Jacques-Olivier Coq, Kazuto Ueda, Shinobu Shimizu, Masahiro Tsuji, Masahiro Hayakawa, and Yoshiaki Sato

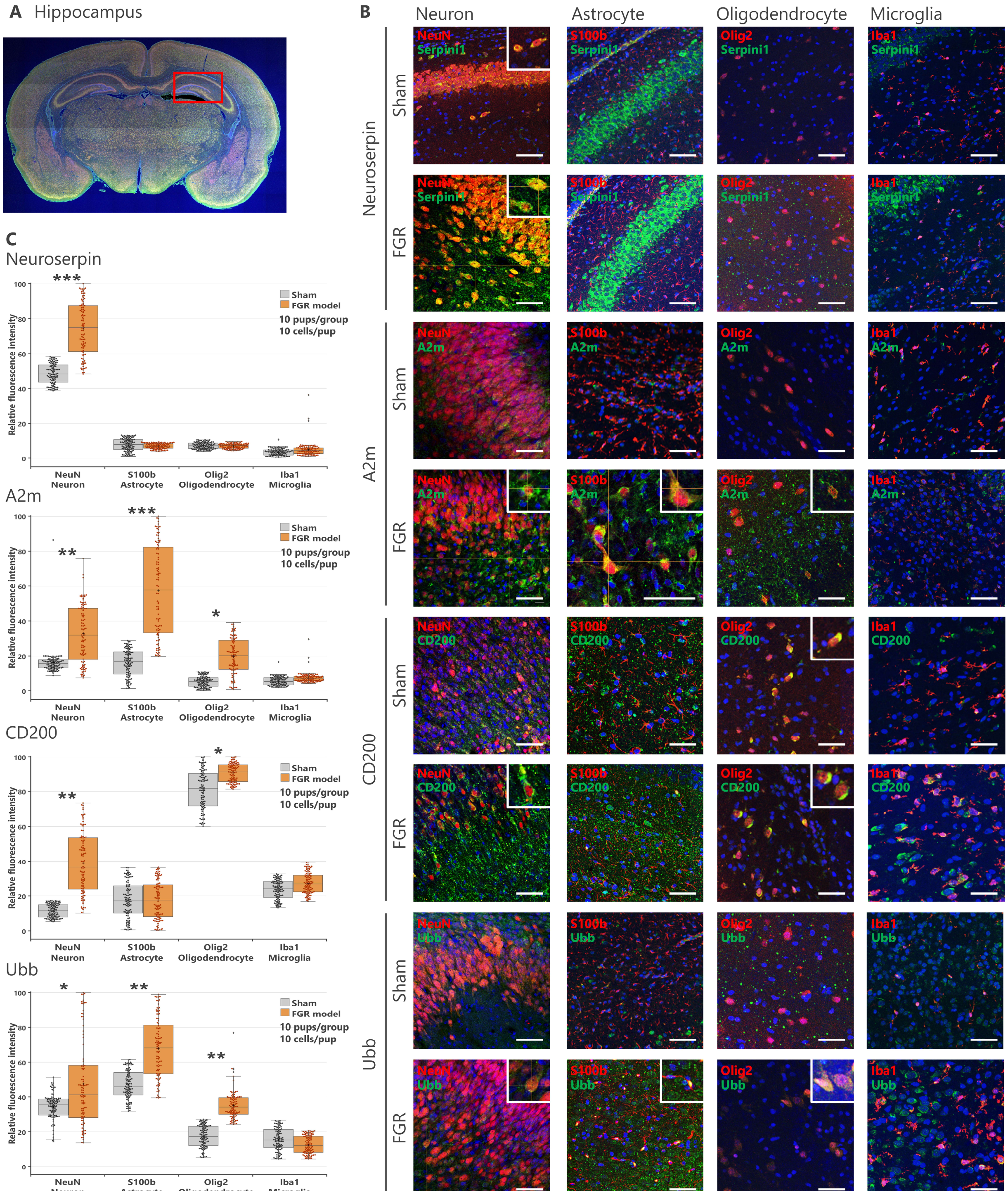

Figure S1. Localization and expression intensity of biomarker candidates in the hippocampus, related to Figure 4. (A) Observed brain regions. (B) Representative images showing A2m, CD200, Ubb, and Neuroserin in NeuN, S100b, Olig2, and Iba1 positive cells. Enlarged images of co-localized cells are shown in the upper right corner of each photo. Scale bar: 50 μm. (C) Graphs showing the relative fluorescence intensity of biomarker candidates in each cell (n = 10/group, totaling 100 cells in each group, ***$p < 0.001$, **$p < 0.01$, *$p < 0.05$). The highest intensity value was set at 100, and the background was set at 1.

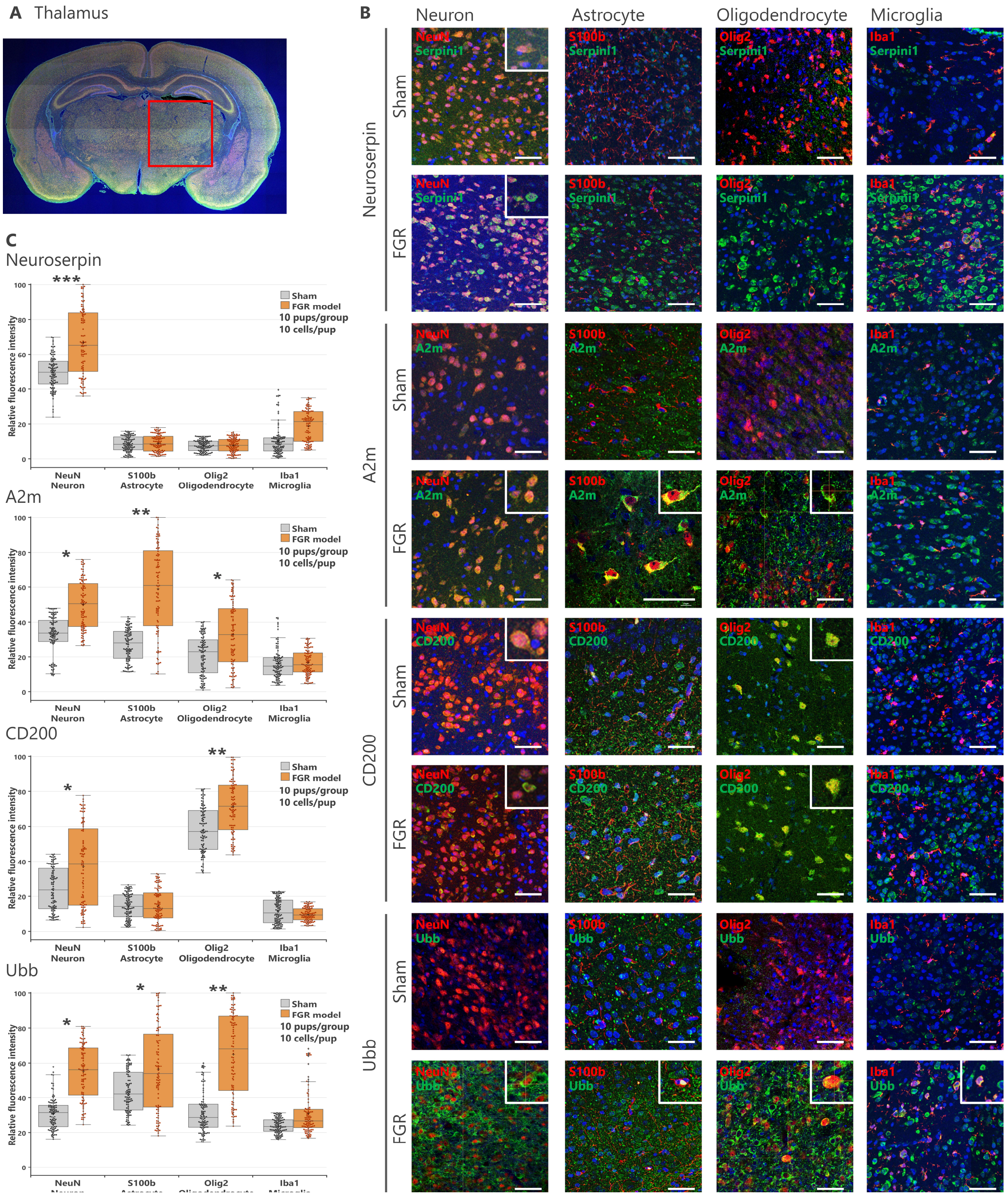

Figure S2. Localization and expression intensity of biomarker candidates in the thalamus, related to Figure 4. (A) Observed brain regions. (B) Representative images showing A2m, CD200, Ubb, and Neuroserin in NeuN, S100b, Olig2, and Iba1 positive cells. Enlarged images of co-localized cells are shown in the upper right corner of each photo. Scale bar: 50 μm. (C) Graphs showing the relative fluorescence intensity of biomarker candidates in each cell (n = 10/group, totaling 100 cells in each group, ***p < 0.001, **p < 0.01, *p < 0.05). The highest intensity value was set at 100, and the background was set at 1.

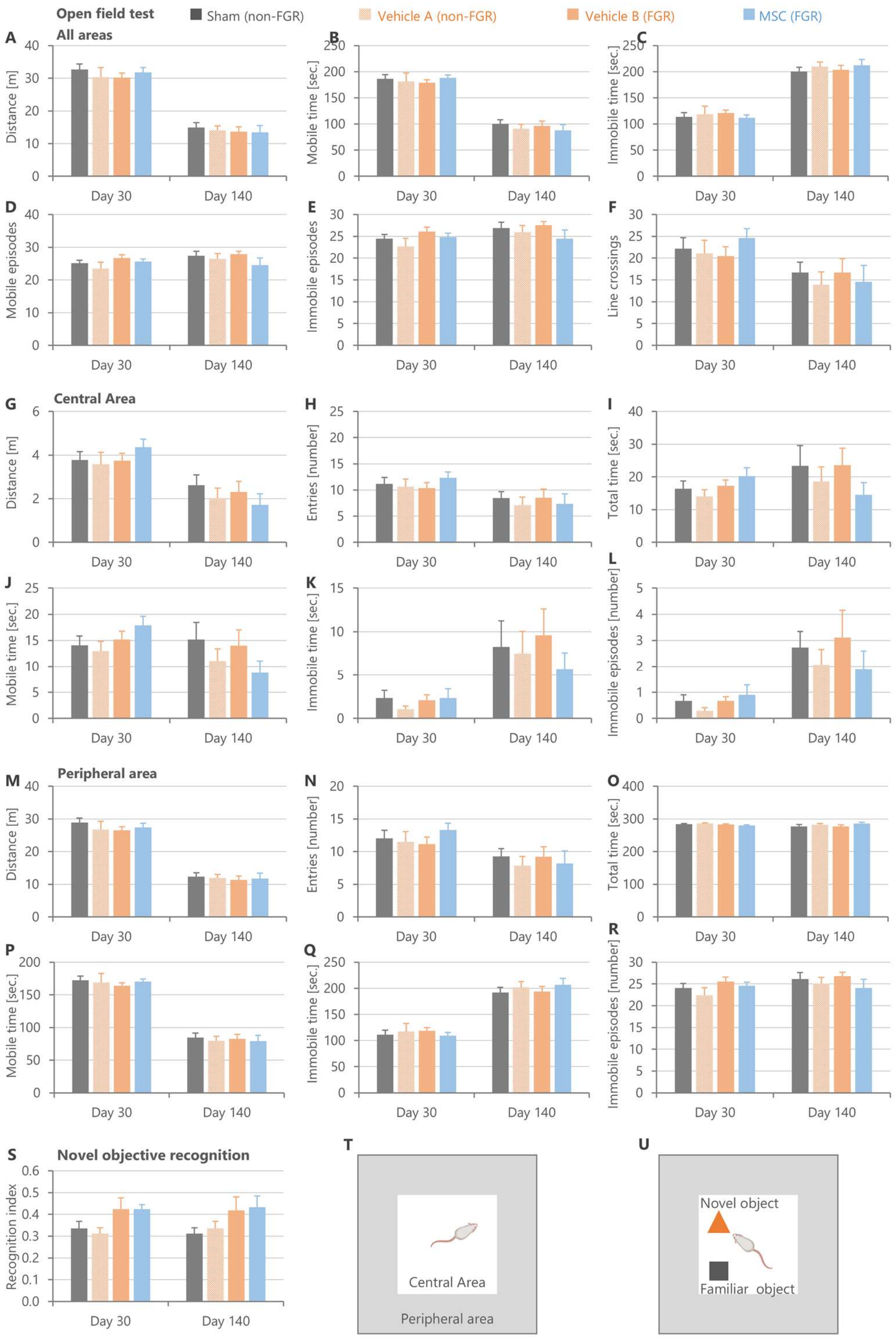

Figure S3. Results of behavioral tests showing no changes due to fetal growth restriction, related to Figure 5. (A-R) Results of the open field test performed on postnatal days 30 and 140. This includes distance traveled, mobile time, immobile time, mobile episodes, immobile episodes, line crossings, and the number of entries into central and peripheral areas (n = 10/group). (S) Results of the novel object recognition test performed on postnatal days 30 and 140 (n = 10/group). (T, U) Schematic depiction of the open field test and novel object recognition test.
